# Targeted Synthesis of End-On Dinitrogen-Bridged Lanthanide Metallocenes and Their Reactivity as Divalent Synthons

**DOI:** 10.1021/jacs.3c07600

**Published:** 2023-09-01

**Authors:** Arpan Mondal, Christopher G. T. Price, Jinkui Tang, Richard A. Layfield

**Affiliations:** † Department of Chemistry, School of Life Sciences, University of Sussex, Brighton BN1 9QJ, U.K.; ‡ State Key Laboratory of Rare Earth Resource Utilization, Changchun Institute of Applied Chemistry, Chinese Academy of Sciences, Changchun 130022, P.R. China

## Abstract

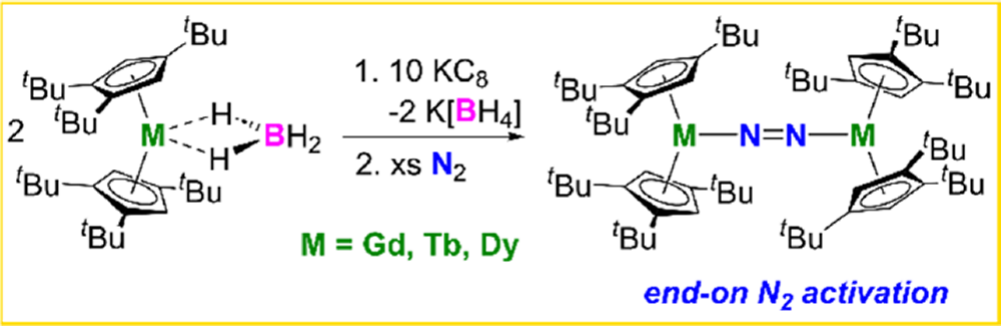

High-yield syntheses of the lanthanide dinitrogen complexes [(Cp_2_^ttt^M)_2_(μ-1,2-N_2_)] (**1_M_**, M = Gd, Tb, Dy; Cp^ttt^ = 1,2,4-C_5_^*t*^Bu_3_H_2_), in which the [N_2_]^2–^ ligands solely adopt the rare end-on or 1,2-bridging mode, are reported. The bulk of the *tert*-butyl substituents and the smaller radii of gadolinium, terbium, and dysprosium preclude formation of the side-on dinitrogen bonding mode on steric grounds. Elongation of the nitrogen-nitrogen bond relative to N_2_ is observed in **1_M_**, and their Raman spectra show a major absorption consistent with N=N double bonds. Computational analysis of **1_Gd_** identifies that the local symmetry of the metallocene units lifts the degeneracy of two 5d_π_ orbitals, leading to differing overlap with the π* orbitals of [N_2_]^2–^, a consequence of which is that the dinitrogen ligand occupies a singlet ground state. Magnetic measurements reveal antiferromagnetic exchange in **1_M_** and single-molecule magnet (SMM) behavior in **1_Dy_**. Ab initio calculations show that the magnetic easy axis in the ground doublets of **1_Tb_** and **1_Dy_** align with the {M–N=N–M} connectivity, in contrast to the usual scenario in dysprosium metallocene SMMs, where the axis passes through the cyclopentadienyl ligands. The [N_2_]^2–^ ligands in **1_M_** allow these compounds to be regarded as two-electron reducing agents, serving as synthons for divalent gadolinium, terbium, and dysprosium. Proof of principle for this concept is obtained in the reactions of **1_M_** with 2,2′-bipyridyl (bipy) to give [Cp_2_^ttt^M(κ^2^-bipy)] (**2_M_**, M = Gd, Tb, Dy), in which the lanthanide is ligated by a bipy radical anion, with strong metal–ligand direct exchange coupling.

## Introduction

Activation of dinitrogen by strongly reducing metal complexes and the subsequent functionalization of N_2_-derived ligands are reactions of considerable importance, both from a fundamental perspective and because of the insight they provide into a variety of biological and catalytic processes.^1,2^ Dinitrogen activation by f-elements has been studied for more than a century in the context of ammonia synthesis, with cerium and uranium reportedly being competent catalysts in the Haber-Bosch process.^3^ More recently, the activation of dinitrogen by well-defined uranium complexes has been demonstrated and, subsequently, used as the basis of non-catalytic routes to ammonia and to other *N*-functionalized species.^4−9^ Complexes of rare-earth elements in the divalent oxidation state^10−14^ and various combinations of trivalent rare earth complexes with alkali metals^15−17^ also show a propensity to activate dinitrogen via reduction, but subsequent conversion of [N_2_]^2–^ into hydrazido derivatives has only been accomplished in very few instances.^18,19^

**Scheme 1:**
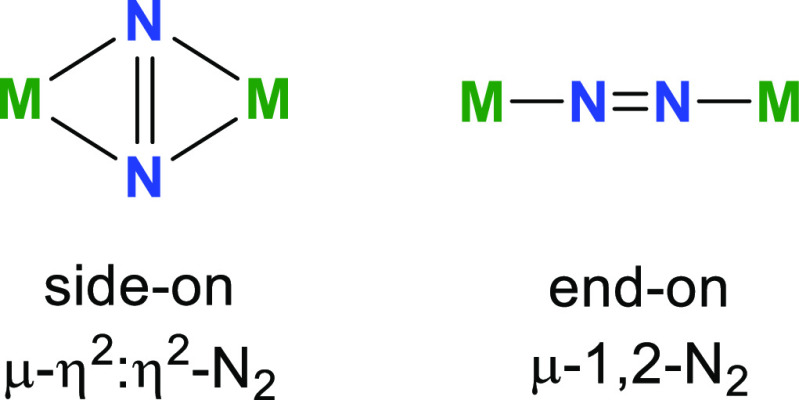
Dinitrogen Coordination Modes toward Rare-Earth Metals, M

Much of the fascination with dinitrogen activation by lanthanides stems from the coordination mode of the N_2_-derived ligand, which can influence the balance of 4f vs 5d orbital usage by the metal. The bridging side-on coordination mode {M_2_(μ-η^2^:η^2^-N_2_)} is common with rare-earth metals, whereas the end-on variant {M_2_(μ-1,2-N_2_)} is uncommon (Scheme 1).^1^ The difference in coordination mode matters since it impacts the electronic structure of the metal and the dinitrogen ligand, while also influencing the stability of the complex.

To date, examples of pure end-on dinitrogen-bridged rare-earth complexes are limited to the bulky amido-ligated anions [{(N″)_3_M}_2_(μ-1,2-N_2_)]^2–^ (N″ = N(SiMe_3_)_2_, M = Sc, Y, Tb), which form as salts of crown- or cryptand-ligated alkali metal cations.^20^ The gadolinium and dysprosium analogues form in the solid state as mixtures of two disordered components, defined by end-on and side-on coordination of the [N_2_]^2–^ ligand.^21^ The scandium complex was found to eliminate N_2_ on irradiation with UV light at −78 °C, and the gadolinium and terbium versions were reported to eliminate N_2_ above −35 °C. The delicate energetic balance between the two coordination modes of [N_2_]^2–^ was underscored by the neodymium congener, which forms as [{(N″)_2_Nd}_2_(μ-1,2-N_2_)]^2–^ below −90 °C and converts into the side-on version [{(N″)_2_Nd}_2_(μ-η^2^:η^2^-N_2_)]^2–^ in the solid-state at higher temperatures.^22^

The end-on bonding mode of dinitrogen has hitherto not been observed in lanthanide metallocene chemistry, whereas lanthanide metallocenes with side-on bridging dinitrogen are known with [N_2_]^2–^ and its *S* = 1/2 radical [N_2_]^3–^ derivative.^23^ We were motivated to target end-on dinitrogen-bridged lanthanide metallocenes to gain insight into how {Cp_2_Ln} fragments influence the electronic structure of the μ-1,2-N_2_ ligand and phenomena such as magnetic exchange interactions and slow magnetic relaxation. Furthermore, by regarding the [N_2_]^2–^ ligand as a two-electron reservoir, the dinitrogen complexes themselves might also serve as surrogate divalent reducing agents for lanthanides that do not readily form the oxidation state +2, allowing them to be used in the synthesis of lanthanide complexes of molecular magnets with radical ligands.

## Results

**Scheme 2:**
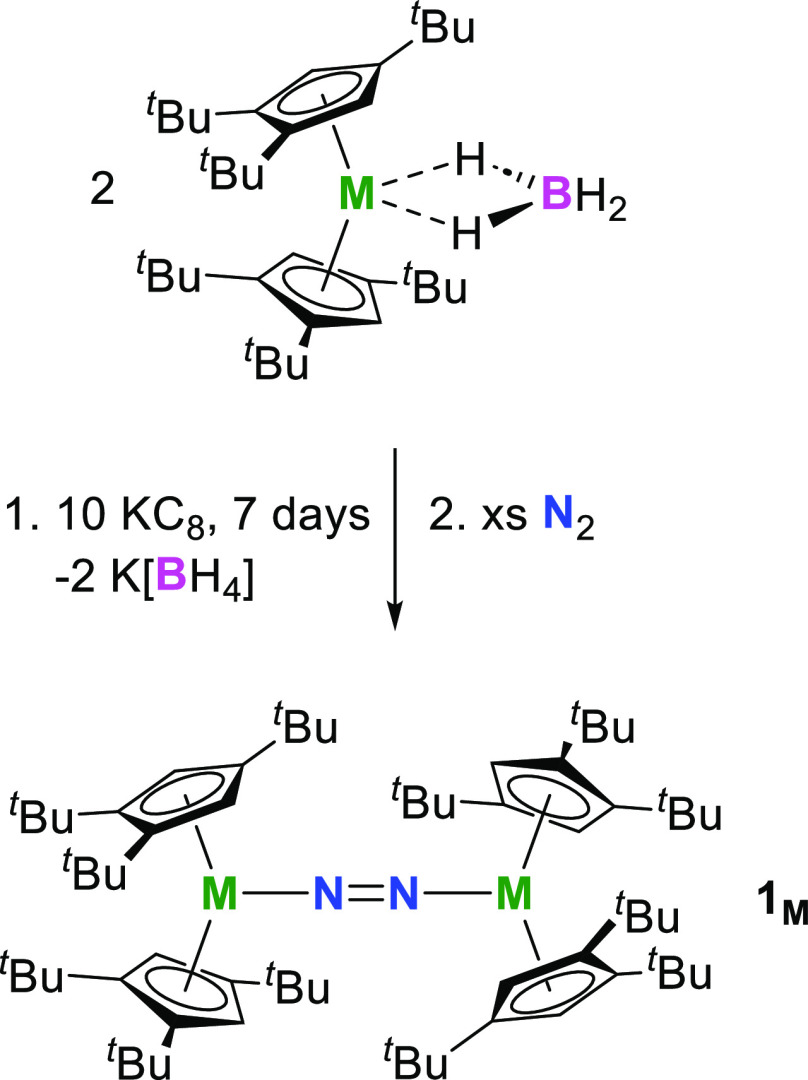
Synthesis of **1_M_** (M = Gd, Tb, Dy)

Our strategy focused on synthesizing [(Cp_2_^ttt^M)_2_(μ-1,2-N_2_)] (**1_M_**, M = Gd, Tb, Dy; Cp^ttt^ = 1,2,4-C_5_^*t*^Bu_3_H_2_) by reduction of [Cp_2_^ttt^M(κ^2^-BH_4_)] with an excess of potassium graphite under an atmosphere of dinitrogen at room temperature. Combined with the relatively small radii of the lanthanide M^3+^ cations, the bulky cyclopentadienyl ligand Cp^ttt^ was selected assuming that the *tert*-butyl substituents would hinder close approach of the two metallocene units, discouraging formation of the side-on dinitrogen coordination mode and encouraging formation of the end-on mode. The target compounds **1_Gd_**, **1_Tb_**, and **1_Dy_** were isolated in yields of 87, 83, and 84%, respectively, based on the lanthanide according to Scheme 2.

In synthesizing **1_M_**, the initial step probably involves reduction of the trivalent metallocenes [Cp_2_^ttt^M(κ^2^-BH_4_)] to divalent [Cp_2_^ttt^M], which then rapidly activates N_2_ by electron transfer. The formation of divalent lanthanide intermediates is reasonable given the similarities between our reaction conditions and those used to prepare other, related divalent lanthanide metallocenes.^14,24,25^ Attempts at isolating the putative [Cp_2_^ttt^M] complexes were made by conducting the KC_8_ reductions under an atmosphere of argon; however, the dinitrogen complexes **1_M_** still form even under these conditions, implying that the divalent metallocenes are sufficiently reactive to scavenge trace N_2_ from the high-purity argon.

**Figure 1:**
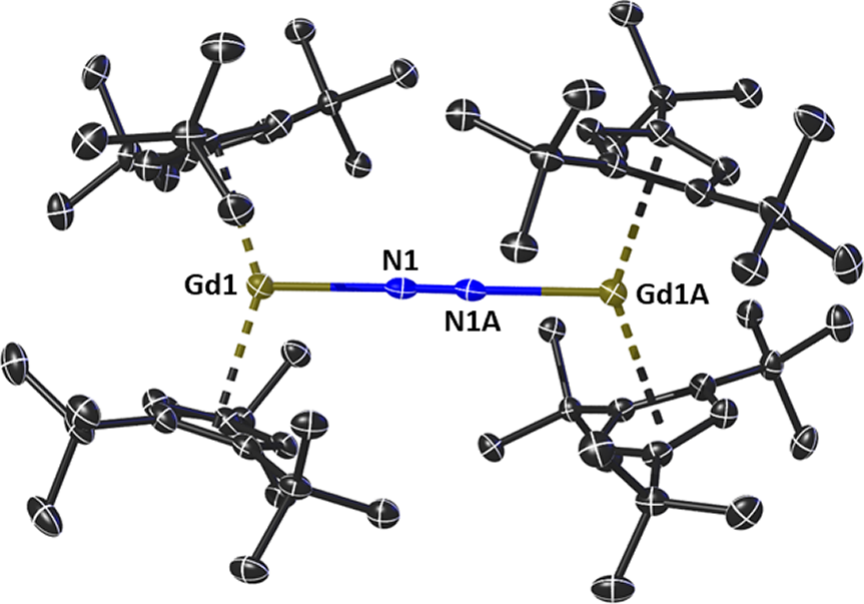
Thermal ellipsoid representation (30% probability) of the molecular structure of **1_Gd_**. For clarity, hydrogen atoms are not shown.

The molecular structures of **1_M_** were determined by single-crystal X-ray diffraction measurements (Figures 1, S1, Table S1). The three complexes are isostructural, forming as centrosymmetric molecules with two {Cp_2_^ttt^M} units bridged by an end-on dinitrogen ligand, and with the inversion center coinciding with the midpoint of the N=N bond. The resulting {M–N=N} connectivities are essentially linear at 179.0(3)°, 179.6(3)°, and 179.3(5)° for **1_Gd_**, **1_Tb_**, and **1_Dy_**, respectively. Consistent with the gradual decrease in ionic radius of the M^3+^ cations, the M–N distances decrease from 2.325(4) Å in **1_Gd_** to 2.296(4) Å in **1_Tb_** and 2.268(7) Å in **1_Dy_**. Concomitantly, the N=N distances increase across the series, being 1.130(8), 1.175(8), and 1.215(13) Å for **1_Gd_**, **1_Tb_**, and **1_Dy_**, respectively. The trend in M–N distances, combined with the apparent flexibility of the N=N bond, produces similar M···M separations of 5.7791(9), 5.7659(9), and 5.7500(9) Å for **1_Gd_**, **1_Tb_**, and **1_Dy_**, respectively. Other pertinent geometric parameters include M–Cp^ttt^ distances of 2.4370(16) and 2.4399(17) Å for **1_Gd_**, 2.4146(19) and 2.419(2) Å for **1_Tb_**, and 2.405(3) and 2.406(3) Å for **1_Dy_**, with associated Cp^ttt^–M–Cp^ttt^ angles of 144.63(8)°, 143.96(9)°, and 143.86(13)°, respectively.

**Figure 2:**
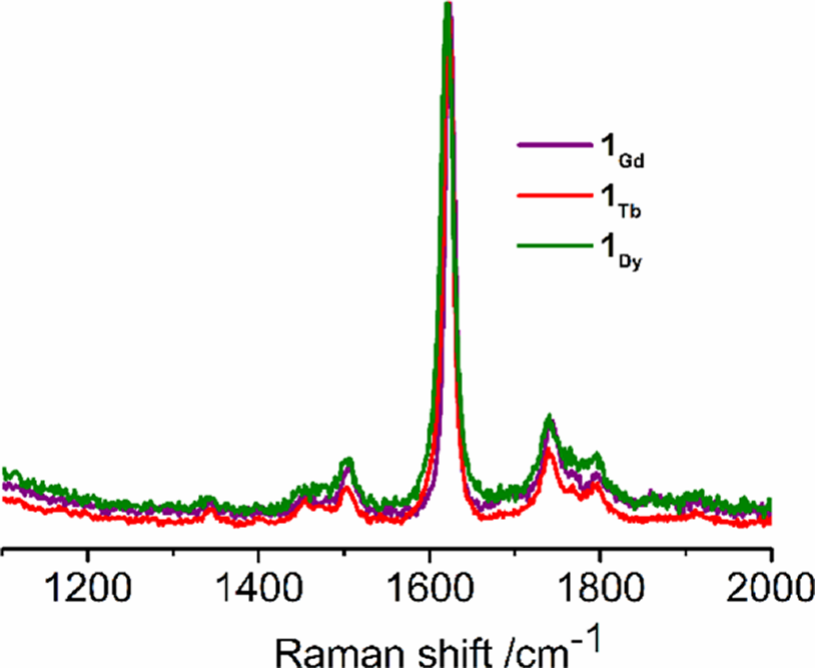
Raman spectra of **1_Gd_**, **1_Tb_**, and **1_Dy_**. Spectra are normalized and the baselines are corrected.

Relative to dinitrogen itself, in which the triple bond length is 1.098 Å, considerable lengthening of the nitrogen–nitrogen bond has occurred upon reduction of N_2_ to give **1_M_**. The extent to which the N_2_ bond lengthens is, evidently, dependent on the lanthanide, although the supporting Cp^ttt^ ligands may also influence this aspect given that the analogous distances in the series [{(N″)_2_M}_2_(μ-1,2-N_2_)]^2–^ are typically longer almost regardless of the metal.^20−22^ However, it has been pointed out that the nitrogen–nitrogen distance is an imperfect measure of the extent of activation^1^ and that greater insight can be obtained from vibrational spectroscopy. The Raman spectra of **1_M_** were therefore recorded and found to be very similar (Figure 2), consisting of dominant signals at 1623, 1621, and 1618 cm^–1^ for **1_Gd_**, **1_Tb_**, and **1_Dy_**, respectively, indicative of N=N double bonds in **1_M_**.^26^

### Analysis of Bonding in **1_Gd_**

**Figure 3:**
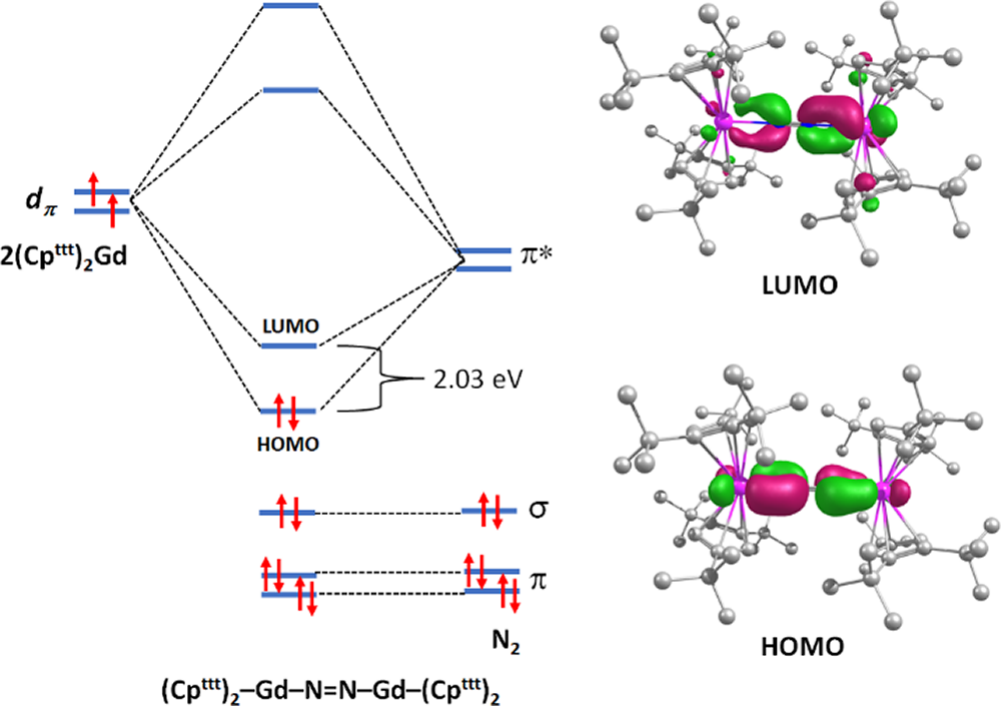
Frontier molecular orbitals in **1_Gd_**.

To understand the bonding within the {M_2_N_2_} core of **1_M_**, we analyzed **1_Gd_** using density functional theory (DFT) calculations, which were performed on the coordinates obtained from the X-ray structure without optimization, except for the hydrogen atoms. Full computational details are provided in the Supporting Information. The calculations show that the highest occupied molecular orbital (HOMO) corresponds to gadolinium-N_2_ interactions and is doubly occupied, indicating transfer of two electrons from two putative [Cp_2_^ttt^Gd] complexes to dinitrogen (Figure 3).

**Figure 4:**
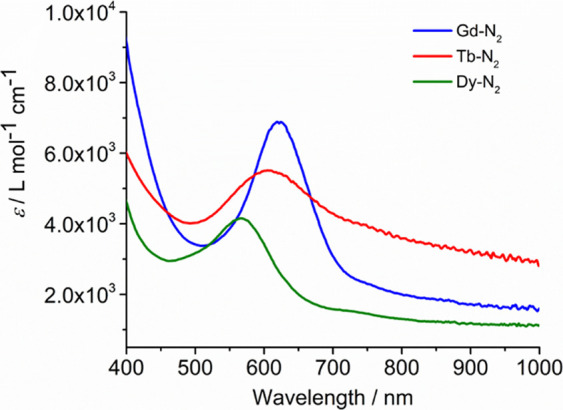
UV–vis spectra in toluene for **1_Gd_** (λ_max_ = 619 nm), **1_Tb_** (λ_max_ = 605 nm), and **1_Dy_** (λ_max_ = 562 nm) at −78 °C.

The π-character of the HOMO arises from overlap between a gadolinium 5d orbital and a π* orbital of the N_2_ ligand. The lowest unoccupied molecular orbital (LUMO) involves an interaction between the other N_2_ π* orbital and a gadolinium 5d orbital, but this is weaker due to the relatively poor spatial overlap. An important consequence of this orbital scenario is the HOMO–LUMO gap of 2.03 eV (approximately 196 kJ mol^–1^), which results in a well-isolated singlet ground state for the bridging [N_2_]^2–^ ligand. The HOMO–LUMO gap calculated for **1_Gd_** agrees well with the observation of a major absorption in the UV-vis spectrum of **1_Gd_** at λ_max_ = 619 nm (Figure 4), corresponding to the HOMO–LUMO transition.

The singlet character of the end-on dinitrogen ligand in **1_Gd_** is in stark contrast with the triplet character of the analogous ligand in [{(N″)_2_M}_2_(μ-1,2-N_2_)]^2–^, which is a consequence of the degeneracy of the π* orbitals being preserved.^20^ In addition, negligible spin density ( (N) = −0.07) was also calculated for the dinitrogen ligand in **1_Gd_** along with a total spin expectation value of ⟨*S*^2^⟩ = 56 for the molecule, implying a high-spin configuration with 14 unpaired electrons. These results further support the singlet configuration of [N_2_]^2–^ in **1_Gd_**. The calculations also reveal that the Gd–N bonds have a 14% weighting of gadolinium, which is composed of 90% 5d character and 9.5% 6p character, with a negligible contribution from the 4f orbitals (Tables S5 and S6). Furthermore, the natural electronic configuration of the gadolinium atoms assigned by the calculations is 4f^6.995^ 5d^0.45^, which equates to the expected 4f^7^ 5d^0^ configuration of Gd^3+^ and agrees with the formal dianionic charge of the dinitrogen ligand. The X-band EPR spectrum of polycrystalline **1_Gd_** at 298 K shows extensive fine structure (Figure S5), and a fit of the experimental spectrum was possible with the inclusion of a small rhombic term with the parameters *g* = 2.001, *D* = −0.014 cm^–1^, and *E* = −0.0013 cm^–1^ (*E*/*D* = 0.09). The EPR data are consistent with previously reported Gd^3+^ complexes of diamagnetic ligands.^27^

### Reactivity of Lanthanide Dinitrogen Complexes

**Scheme 3:**
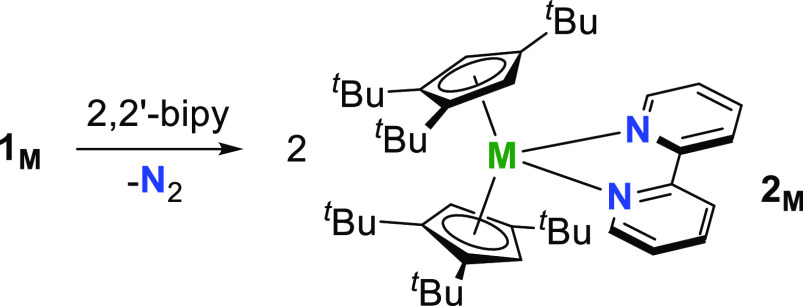
Synthesis of **2_M_** (M = Gd, Tb, Dy)

The strongly reducing nature of [N_2_]^2–^ has considerable potential to allow **1_M_** to be developed for bespoke electron-transfer reactivity. For example, adding the two-electron reducing agents **1_M_** to *N*-heterocyclic ligands with an odd number of binding sites should result in electron transfer and elimination of N_2_, accompanied by the formation of paramagnetic lanthanide metallocene complexes by heterocyclic radical anions. Achieving reactivity of this nature would provide a new method for the synthesis of molecular magnets such as SMMs, going beyond early studies examining the reactivity of diamagnetic side-on dinitrogen complexes of lanthanum and lutetium.^28,29^ Indeed, it is noteworthy that bimetallic lanthanidocene complexes of radical *N*-donor ligands are a well-known type of SMM,^30−35^ with some examples displaying remarkable magnetic hysteresis properties arising from direct magnetic exchange with the radical ligand.^23,36,37^ To the best of our knowledge, monometallic lanthanide metallocene SMMs containing radical ligands are unknown. We therefore undertook to synthesize such species by reacting **1_M_** with 2,2′-bipyridyl (bipy), resulting in the formation of [Cp_2_^ttt^M(κ^2^-bipy)] (**2_M_**, M = Gd, Tb, Dy) according to Scheme 3.

**Figure 5:**
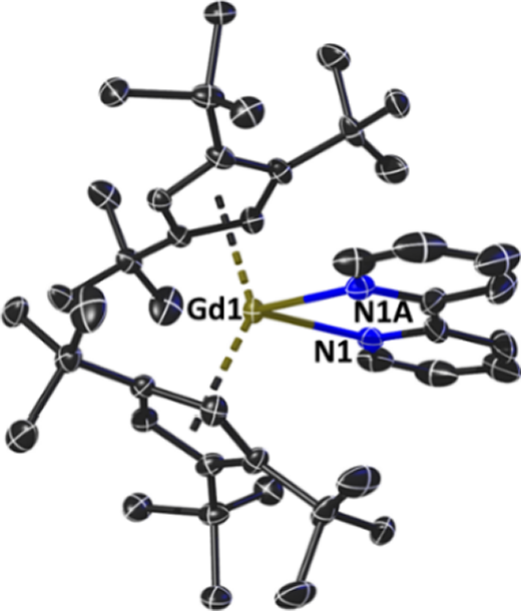
Thermal ellipsoid representation (50% probability) of the molecular structure of **2_Gd_**. For clarity, hydrogen atoms are not shown.

Complexes **2_M_** were isolated in excellent yields of 90, 95, and 82% for **2_Gd_**, **2_Tb_**, and **2_Dy_**, respectively. X-ray crystallography confirmed that **2_M_** adopt bent metallocene structures with a κ^2^-bipy ligand and a mirror plane bisecting the molecules through the metal, oriented perpendicular to the bipy plane (Figures 5, S2 and Tables S2 and S4). The M–N distances of 2.4052(18), 2.384(3), and 2.365(2) Å in **2_Gd_**, **2_Tb_**, and **2_Dy_**, respectively, are much longer than the related distances in **1_M_** to accommodate the larger bipy ligand. In response, the M–Cp^ttt^ distances increase by about 0.07 Å relative to **1_M_** to 2.5036(10), 2.4832(14), and 2.4650(11) Å in **2_Gd_**, **2_Tb_**, and **2_Dy_**, respectively, and the associated Cp^ttt^–M–Cp^ttt^ angles narrow to 140.17(5)°, 141.04(7)°, and 141.28(5)°.

**Figure 6:**
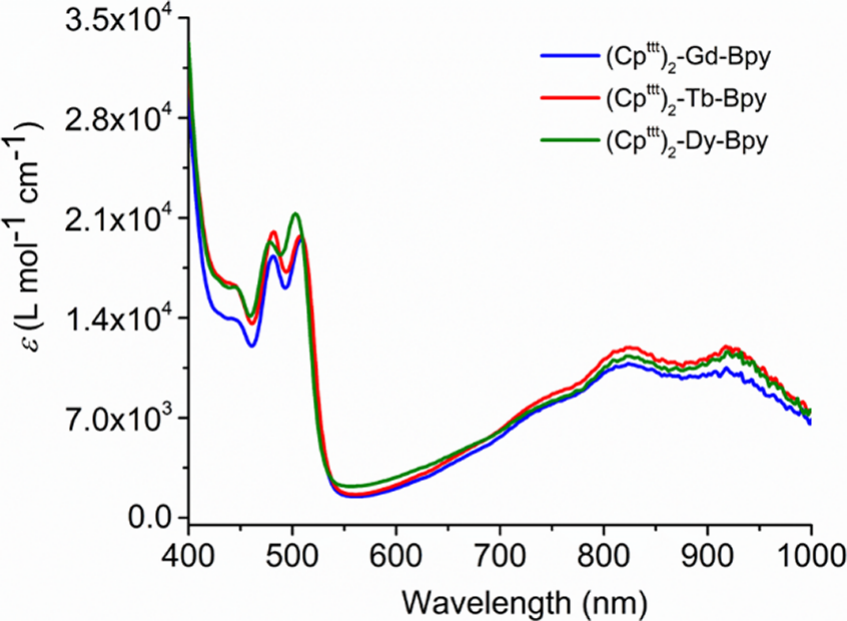
UV–vis spectra in hexane for **2_Gd_**, **2_Tb_**, and **2_Dy_** complexes at room temperature.

In contrast to **1_Gd_**, the X-band EPR spectrum of polycrystalline **2_Gd_** at 298 K consists of a single-line absorption at *g* = 1.98, consistent with the presence of 2,2′-bipy in its *S* = 1/2 radical anion form (Figure S6). The UV–vis spectra of **2_M_** which are virtually identical, contain more features than the analogous spectra of **1_M_**, including a series of overlapping absorptions in the region 650–1000 nm and higher-energy transitions below approximately 500 nm (Figure 6). A time-dependent DFT (TD-DFT) analysis of the UV–vis spectrum of **2_Gd_** revealed that the group of lower-energy transitions is a combination of ligand-to-metal charge transfer from π* orbitals of the bipy radical anion to gadolinium 5d orbitals and intra-ligand transitions within the π/π* manifold of [bipy]^−^ (Figure S7, Table S7). The higher-energy transitions occur solely within the [bipy]^−^ orbital manifold.

Lanthanide complexes of reduced bipyridyl and related *N*-heterocyclic ligands are an established class of compounds, yet examples containing metals other than ytterbium or samarium are rare.^38,39^ The popularity of ytterbium stems from the ready availability of ytterbium(II) metallocenes,^40−45^ especially [(C_5_Me_5_)_2_Yb], which has been used to reduce *N*-heterocycles to give complexes such as [(C_5_Me_5_)_2_Yb(κ^2^-bipy)], which has an unusual electronic ground state with intermediate valence and multiconfigurational character. The rich redox chemistry of bipy-type ligands has also been showcased with trivalent and tetravalent actinide complexes typified by [Cp_2_^ttt^An(κ^2^-bipy)] (trivalent An = U, tetravalent An = Th, U), which were synthesized by in situ reduction of bipy with KC_8_ in the presence of [Cp_2_^ttt^AnCl_2_].^46−48^ Actinide metallocenes such as these are notable for their reactivity as one- or two-electron reducing agents in a range of small-molecule activation processes. Similar synthetic routes were also developed for the potassium salt of a radical diazafluorenylidene-substituted phospha-alkene and a boryl-substituted bipy radical, which were used in salt metathesis reactions to give complexes with the radical ligand bound to gadolinium, terbium, and dysprosium.^49,50^

The availability of **1_M_** as bespoke reducing agents offers numerous synthetic advantages. For example, complexes of **1_M_** allow an extension of lanthanide-based reduction chemistry beyond the classical divalent lanthanides samarium, europium, and ytterbium. In place of large excesses of KC_8_ as the reductant, the dinitrogen ligand in **1_M_** is a well-defined two-electron source that converts into N_2_ as a traceless leaving group following reduction, avoiding formation of salt-like bimetallic products. The high-yielding synthesis of **1_M_** is also noteworthy.

### Magnetic Properties

The magnetic properties of lanthanide complexes with end-on bridging dinitrogen ligands have, hitherto, not been subjected to detailed investigations by DC and AC susceptometry. To address this gap in understanding, the temperature dependence of the molar magnetic susceptibility () in an applied field of 1 kOe and the field dependence of the magnetization (*M*) in fields of 0–70 kOe were measured for all compounds. Additionally, the AC susceptibility and magnetic hysteresis properties of **1_Tb_**, **1_Dy_**, **2_Tb_**, and **2_Dy_** were investigated to check for SMM behavior.

**Figure 7:**
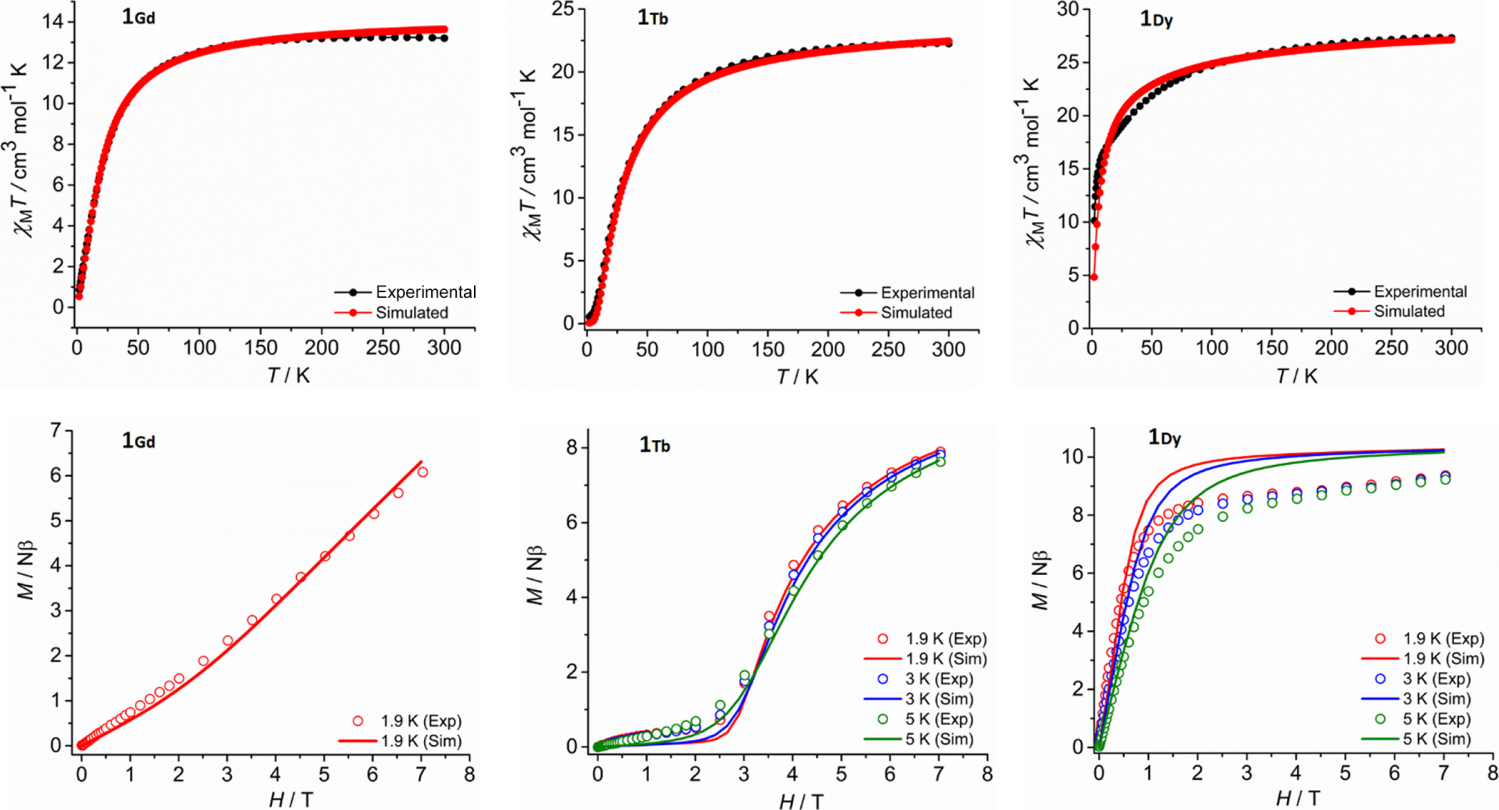
χ_M_*T*(*T*) and *M*(*H*) plots for **1_Gd_**, **1_Tb_**, and **1_Dy_** with simulations according to eqs 1 or 2 in the main text.

The χ_M_*T* value for **1_Gd_** at 300 K is 13.45 cm^3^ K mol^–1^, which is somewhat lower than the theoretical value of 15.75 cm^3^ K mol^–1^ predicted for two non-interacting Gd^3+^ ions with *g* = 2.0 (Figure 7).^51^ On lowering the temperature to 120 K, χ_M_*T* decreases gradually before decreasing rapidly to reach 0.83 cm^3^ K mol^–1^ at 2 K. The overall temperature-dependence of χ_M_*T* for **1_Gd_** is consistent with antiferromagnetic exchange via the end-on dinitrogen ligand. The *M*(*H*) plot for **1_Gd_** at 2 K shows that the magnetization increases in a near-linear manner up to a value of 6.10 Nβ at the maximum field of 70 kOe attainable with the magnetometer, well below the saturation magnetization value of 14 Nβ expected for two Gd^3+^ ions (Figure 7). Simultaneous fits of the susceptibility and magnetization data were achieved using the spin Hamiltonian stated in eq 1 as implemented in the PHI software,^52^ where *J* is the exchange coupling constant, *g*_Gd_ and *S*_Gd_ denote the *g*-tensor and total spin associated with the gadolinium centers, respectively, and β is the Bohr magneton. Using *g* = 1.90, a *J*-value of −0.81 cm^–1^ was obtained for **1_Gd_**.
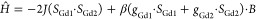
1
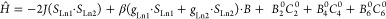
2

At 300 K, χ_M_*T* for **1_Tb_** is 23.13 cm^3^ K mol^–1^, which is slightly lower than the value of 23.64 cm^3^ K mol^–1^ for two non-interacting Tb^3+^ ions.^51^ The gradual decrease in χ_M_*T* as the temperature decreases toward 100 K is followed by a precipitous drop to 0.52 cm^3^ K mol^–1^ at 2 K, most likely due to a combination of antiferromagnetic exchange and depopulation of excited crystal field levels (Figure 7). The *M*(*H*) plots in the temperature range 1.9–5.0 K show a gradual increase in magnetization up to 25 kOe before increasing more rapidly toward 8 Nβ, but without reaching saturation at 70 kOe (Figure 7). The data again indicate antiferromagnetic exchange between the Tb^3+^ centers via the end-on dinitrogen ligand. To simulate the magnetic properties of **1_Tb_**, the SINGLE_ANISO module as implemented in ORCA 5.0.2 was used to compute the *g*-tensors and crystal field parameters of the low-lying excited states within the ^7^F_6_ ground multiplet of Tb^3+^ (Tables S11–S13).^53,54^ The axial crystal field parameters *B*_*k*_^0^ and *C*_*k*_^0^ (*k* = 2,4,6) were then included in the spin Hamiltonian in eq 2, leading to good fits of the χ_M_*T*(*T*) and *M*(*H*) data with *J*= −0.65 cm^–1^ (Figure 7).

The real and imaginary components of the AC susceptibility of **1_Tb_** as functions of temperature, i.e., χ^′^(*T*) and χ^″^(*T*), respectively, were measured in an AC field of 3 Oe and a frequency of 1000 Hz. Slow magnetic relaxation was not observed, and the *M*(*H*) magnetic hysteresis measurements produced closed S-shaped traces at 1.9 K (Figures S8 and S9). Since complexes of the non-Kramers ion Tb^3+^ normally show SMM behavior when occupying a high-symmetry environment or when bound to a radical ligand,^36,55^ the observations on **1_Tb_** are in line with expectations based on magneto-structural correlations developed for lanthanide metallocene SMMs.^56^

The DC susceptibility of **1_Dy_** produced a χ_M_*T* value of 27.27 cm^3^ K mol^–1^ at 300 K, slightly lower than the expected value of 28.34 cm^3^ K mol^–1^ expected for two non-interacting Dy^3+^ ions.^51^ A gradual decrease in the susceptibility on lowering the temperature results in a χ_M_*T* value of 10.13 cm^3^ K mol^–1^ at 2 K (Figure 7), indicating antiferromagnetic exchange and depopulation of excited crystal field levels, as in **1_Tb_**. At 1.9–5.0 K, the magnetization of **1_Dy_** increases rapidly with field up to 10 kOe and then increases more gradually to reach a value of 9.36 Nβ at 70 kOe (Figure 7). A good fit of the susceptibility data was achieved for **1_Dy_** using eq 2 with *g*_Dy_ = 1.33 and the calculated crystal field parameters for Dy^3+^ (^6^H_15/2_ ground state) in Table S11, resulting in an exchange coupling constant of *J* = – 0.07 cm^–1^.

**Figure 8:**
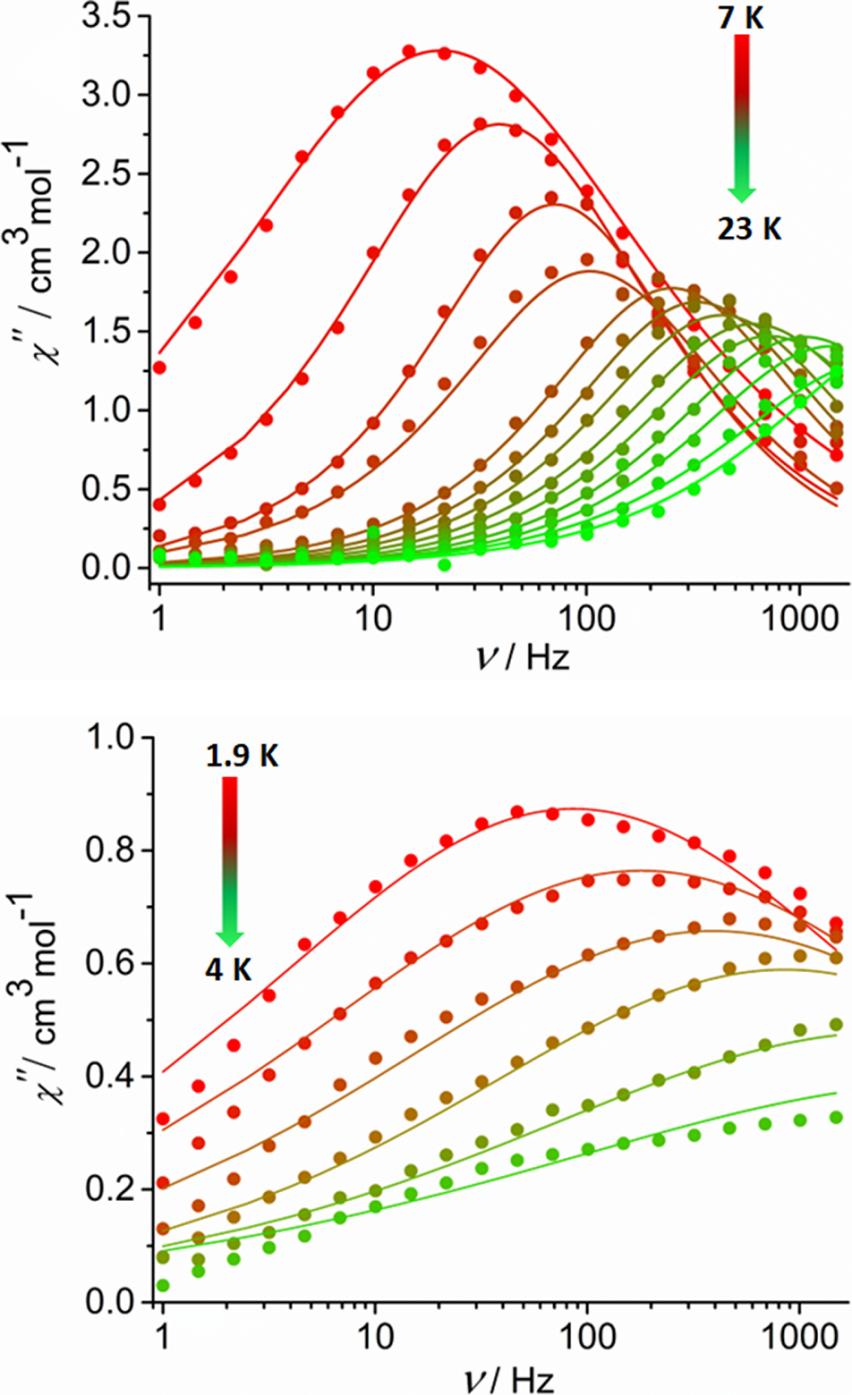
Imaginary part of the AC susceptibility as a function of frequency, χ^″^(ν), in zero DC field for **1_Dy_** at *T* = 7–23 K (upper) and for **2_Dy_** at *T* = 1.9–4.0 K (lower).

In contrast to **1_Tb_**, χ^″^(ν) for **1_Dy_** in zero DC field above 7 K shows maxima up to 23 K, with the maxima shifting to a higher frequency with increasing temperature (Figures 8 and S10). A Cole–Cole plot of χ^″^(χ^′^) was fitted using α-parameters in the range 0.25–0.39, implying a reasonably broad distribution of relaxation times, τ. The plot of ln(τ/*s*) versus *T*^–1^ (Figure S11, Table S9) was fitted with Orbach and Raman terms according to eq 3, where τ_0_ is the attempt time, *U*_eff_ is the effective energy barrier, *C* is the Raman coefficient, and *n* is the Raman exponent.
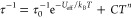
3The resulting fit parameters are τ_0_ = 7.30 × 10^–10^ s, *U*_eff_ = 180(37) cm^–1^, *C* = 0.20(5) s^–1^ K^–*n*^, and *n* = 3.3(3). The observation of SMM behavior in the AC susceptibility is complemented by a narrow opening of the *M*(*H*) hysteresis loop at 1.9 K (Figure S12).

For **2_Gd_**, χ_M_*T* at 300 K is 7.48 cm^3^ K mol^–1^, close to the expected value of 7.50 cm^3^ K mol^–1^ for a Gd^3+^ ion coupled antiferromagnetically to a bipy radical anion with *S* = 1/2. The temperature-dependence of χ_M_*T* decreases steadily down to 75 K before decreasing more rapidly to reach 1.68 cm^3^ K mol^–1^ at 2 K (Figure S13). A gradual increase in the magnetization was observed for **2_Gd_** at 2 K up to a field of approximately 30 kOe, before leveling off at higher fields and reaching 5.93 Nβ at 70 kOe but not saturating (Figure S13). A fit of the susceptibility for **2_Gd_** using eq 4, where *g*_rad_ and *S*_rad_ refer to the *g*-tensor and total spin of the bipy radical anion, was obtained using *g* = 2.0 for Gd^3+^ and the bipy radical anion, yielding an isotropic exchange coupling constant of *J*= −5.50 cm^–1^ (Figure S13). This value is comparable in magnitude to those determined for other gadolinium complexes of radical *N*-heterocyclic ligands.^37,49,50^
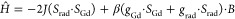
4
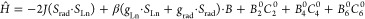
5

The DC susceptibility of **2_Tb_** is similar to that of **1_Tb_**. From a value of 11.43 cm^3^ K mol^–1^ at 300 K, χ_M_*T* decreases gradually down to 100 K before showing a much sharper decrease at lower temperatures and reaching 1.29 cm^3^ K mol^–1^ at 2 K (Figure S14). The room-temperature value of the susceptibility is essentially the same as that of 11.46 cm^3^ K mol^–1^ expected for a Tb^3+^ ion coupled antiferromagnetically to an *S* = 1/2 organic radical. The coupling is also reflected in the *M*(*H*) data at 1.9–5.0 K, with a steady increase in magnetization occurring up to 35 kOe, followed by a sharp upturn at higher fields without approaching saturation at 70 kOe (Figure S14). A reasonable simulation of the χ_M_*T*(*T*) data was obtained for **2_Tb_** using the spin Hamiltonian in eq 5 with *g*_rad_ = 2.0, *g*_Tb_ = 1.51, and the crystal field parameters stated in Table S15, which produced a terbium-bipy exchange coupling constant of *J* = – 4.65 cm^–1^.

While the χ^″^(ν) data for **2_Tb_** do not display maxima (Figure S15), openings in the *M*(*H*) hysteresis loops were observed up to 6 K with a small remanent magnetization in zero field (Figure S16). Bifurcation in the field-cooled/zero-field-cooled (FC/ZFC) susceptibility also occurred at 5.3 K, broadly consistent with SMM behavior (Figure S17). The absence of maxima in the AC susceptibility likely indicates that fast quantum tunneling of the magnetization (QTM) is the dominant relaxation process in **2_Tb_** in zero field, which is corroborated below with the aid of an ab initio theoretical study.

The DC magnetic susceptibility of **2_Dy_** is consistent with a Dy^3+^ ion coupled antiferromagnetically to the bipy radical anion, the value of 13.96 cm^3^ K mol^–1^ for χ_M_*T* at 300 K being close to the expected value of 13.80 cm^3^ K mol^–1^. Lowering the temperature results in the susceptibility reaching 1.51 cm^3^ K mol^–1^ at 2 K (Figure S18). The *M*(*H*) data for **2_Dy_** show an increase in the magnetization in low fields, followed by a plateau around 35 kOe, and then a second rapid increase to reach 3.3 Nβ at 70 kOe, but without saturation (Figure S18). Since the magnetization is clearly still increasing at this field value, relatively strong direct antiferromagnetic exchange should be occurring. A fit of the susceptibility of **2_Dy_** using eq 5 with *g*_rad_ = 2.0 and *g*_Dy_ = 1.36 and the crystal field parameters stated in Table S15 gave *J* = – 3.50 cm^–1^, which is comparable to the isotropic *J*-value determined for **2_Gd_** and the *J*-value for **2_Tb_**. Since *J*-values for highly anisotropic lanthanide-radical pairs are rarely reported, few systems are available for comparison. However, the couplings in **2_Tb_** and **2_Dy_** are seemingly weaker than those found in the [Bi_2_]^3–^ radical bridged dimetallic systems [{C_5_Me_5_)_2_M}_2_(μ-η^2^:η^2^-Bi_2_)]^−^ (M = Tb, Dy),^57^ most likely because of the relatively diffuse spin density in the bipy radical anion in **2_M_**.

SMM behavior was observed for **2_Dy_** in zero DC field, although this is less pronounced than for **1_Dy_**. Broad maxima in χ^″^(ν) occur from 1.9 to 4.0 K (Figures 8 and S19), with the relaxation times allowing a fit of the Cole–Cole plot with α= 0.6–0.7, indicating a broad range of relaxation times (Figure S20, Table S10). A fit of the relaxation times was possible using Orbach and Raman terms according to eq 3, which yielded τ_0_ = 1.40 × 10^–8^ s, *U*_eff_ = 24(5) cm^–1^, *C* = 19(4) s^–1^K^–*n*^, and *n* = 5.2(3). The magnetic hysteresis measurements showed open loops up to 9 K, with two distinct steps around 24 and 59 kOe due to QTM at the magnetic fields corresponding to level crossings (Figure S21). A slight bifurcation of the FC/ZFC data was observed at 7 K (Figure S22).

The energy barrier and hysteresis properties of **1_Dy_** can be interpreted in terms of how the end-on dinitrogen ligand impacts the crystal field at dysprosium relative to the influence of the Cp^ttt^ ligands. It is well-known that large energy barriers and open hysteresis loops can occur in dysprosium metallocene SMMs because the *bis*(cyclopentadienyl) framework provides a dominant axial crystal field.^58−63^ In SMMs with competing equatorial crystal fields such as [{(η^5^-Cp*)_2_Dy}(μ-Fp)]_2_ (Fp = CpFe(CO)_2_),^56^ a large energy barrier of 662 cm^–1^ and wide butterfly hysteresis loops can be observed, albeit with the latter showing negligible coercivity and remnant magnetization. In contrast, the molecular structure of **1_Dy_** reveals that the Dy–N distances to the equatorial anionic nitrogen donor atoms are shorter by almost 0.14 Å than the Dy–Cp centroid distances, suggesting that the end-on bridging dinitrogen ligand should dominate the crystal field. This structural property of **1_Dy_** also explains why the barrier of 180(37) cm^–1^ is much lower than those found in purely axial dysprosocenium SMMs, which can exceed 1500 cm^–1^,^58,60,62,64,65^ and similar to the barriers of approximately 100–300 cm^–1^ found in SMMs of the type [{(η^5^-C_5_H_4_Me)_2_Dy}{μ-ER_*n*_}]_3_ (ER_*n*_ = MesP(H), MesAs(H), MesSb(H), MesSe, Mes = mesityl).^66−68^ The same rationale applies to **1_Tb_**, with the added requirement for strict axial symmetry for non-Kramers ions, hence the poorer SMM properties.

To support the empirical observations on **1_Tb_** and **1_Dy_**, multireference ab initio calculations were used to provide deeper insight into their electronic structure. To simplify the calculations, one of the Ln^3+^ ions in the dinitrogen complexes was replaced by diamagnetic Y^3+^ and the resulting hypothetical heterobimetallic species subjected to the SINGLE_ANISO routine implemented in ORCA 5.0.2. A complete active space (CAS) of CAS(8,7) was used for **1_Tb_** and CAS(9,7) for **1_Dy_**, with the calculations yielding the *g*-tensors and crystal field parameters of the low-lying excited states. Full computational details are provided in the Supporting Information (Tables S11–S14).

**Figure 9:**
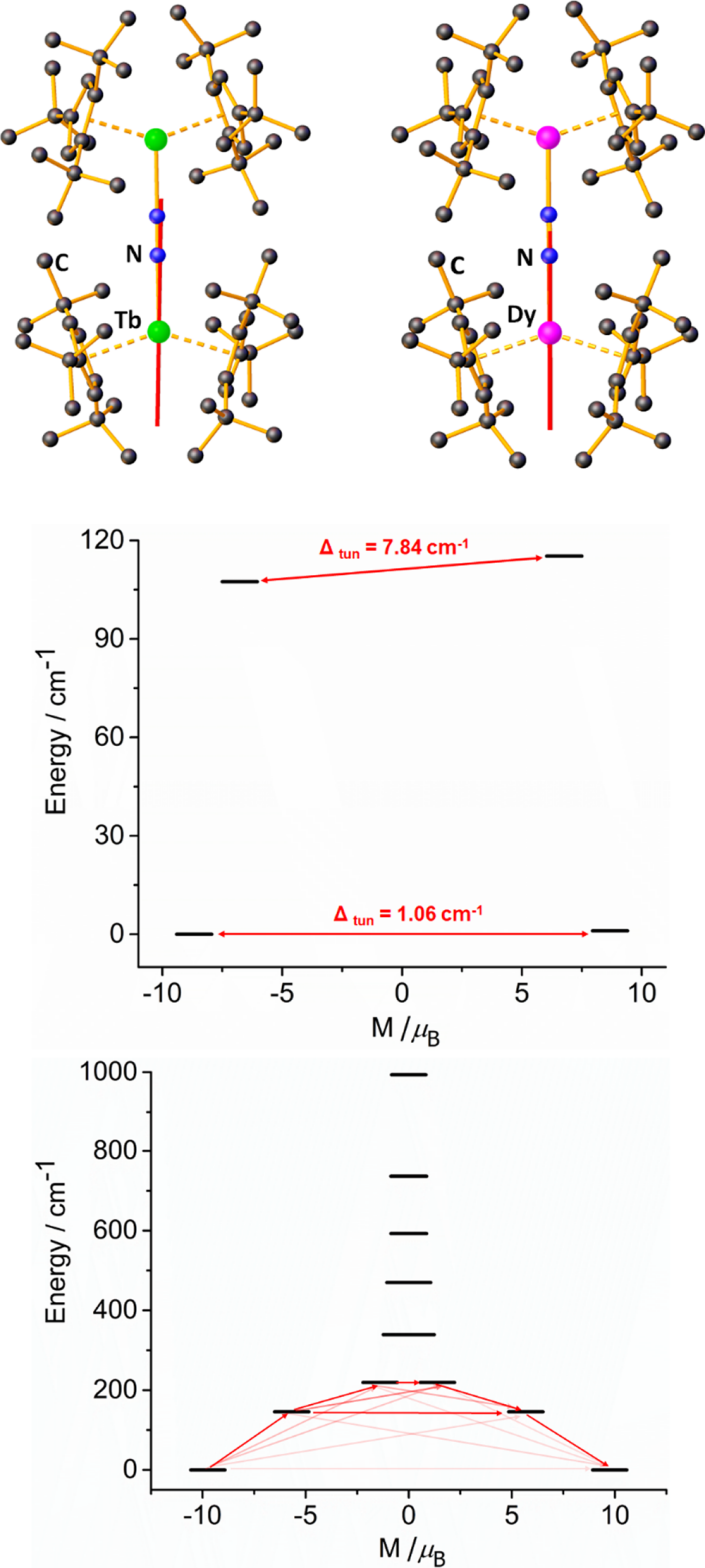
Upper: easy axis of magnetization (red lines) in the ground doublets of **1_Tb_** and **1_Dy_**. Middle: relaxation energy barrier for **1_Tb_**. Lower: relaxation energy barrier for **1_Dy_** showing possible transition as red lines, with darker shading indicating a more probable transition.

The calculations show that the end-on dinitrogen ligand in **1_Tb_** and **1_Dy_** does indeed dominate the crystal field, with the easy axis of magnetization on each lanthanide center coinciding with the {M–N=N–M} axis (Figure 9). This result is a marked contrast to the commonly observed picture with dysprosium metallocene SMMs, where the easy axis in the ground Kramers doublet (KD) typically passes through both cyclopentadienyl ligands.^63,69,70^ Constructing a relaxation energy barrier for **1_Tb_** revealed a tunnel splitting in the ground doublet of Δ_tun_ = 1.062 cm^–1^, which reduces the bistability and is consistent with the poor SMM properties of this species. Similar analysis of **1_Dy_** shows that the ground KD is strongly axial, with *g_x_* = 0.090, *g_y_* = 0.184 and *g_z_* = 19.65, with the wavefunction consisting of 97% |*M_J_*| = 15/2 character. In contrast, the first-excited KD at 146 cm^–1^ has large transverse components with *g_x_* = 2.501, *g_y_* = 5.153, and *g_z_* = 12.90, and is described by a strong admixture of *M_J_* wavefunctions. Since the calculated and experimental energy barriers of 146 and 189(37) cm^–1^, respectively, are comparable in magnitude, Orbach relaxation in **1_Dy_** should proceed via the first-excited KD.

## Discussion

The original rationale for targeting metallocene-capped lanthanide complexes of end-on bridging [N_2_]^2–^ invoked the idea of steric bulk to preclude formation of the common side-on bonding mode. Since side-on N_2_-bridged di-lanthanide metallocenes with Ln = Gd, Tb or Dy tend to be capped by relatively small C_5_Me_5_ or C_5_Me_4_H ligands,^23^ the bulk of Cp^ttt^ is evidently sufficient for the end-on bridging to form exclusively in **1_M_**. The same dinitrogen bonding mode is therefore also likely to be observed in analogous complexes of heavier, smaller lanthanides beyond dysprosium. For the lighter lanthanides with larger radii, it has been shown that the bonding mode of dinitrogen is finely balanced between the two possibilities by steric and other factors, with both end-on and side-on occurring. It is, therefore, of interest to explore the limits and applicability of the chemistry developed for **1_M_** across the full range of the lanthanide series in future work.

The switch from bulky *bis*(trimethylsilyl)amido as the capping ligand in the dinitrogen-bridged di-lanthanide complexes studied by Evans and co-workers^20−22^ to Cp^ttt^ in **1_M_** has important consequences for the interactions of the lanthanide valence 5d orbitals and [N_2_]^2–^ and, hence, the spin configuration of the dinitrogen ligand. The local symmetry of the lanthanide sites results in effective overlap of a 5d_π_ orbital with one of the dinitrogen π* orbitals, and poorer overlap of a second 5d_π_ orbital with the other dinitrogen π* orbital. The resulting HOMO–LUMO gap produces an energetically isolated singlet configuration for the end-on bridging [N_2_]^2–^ ligand in **1_M_**, whereas a triplet configuration was proposed for the end-on dinitrogen complexes capped by *bis*(trimethylsilyl)amido ligands. These contrasting scenarios point toward the possibility of engineering the singlet-triplet energy gap via the capping ligand bound to the lanthanide, which could be used to address magnetic and photophysical properties of lanthanide metallocenes.

The ability to synthesize and isolate **1_M_** in yields reproducibly greater than 80% offers opportunities for new lanthanide-based reduction chemistry without the need to use KC_8_ in the presence of the target substrate, i.e., 2,2′-bipy in the case of **2_M_**. This is especially valuable for lanthanides that do not readily form a stable +2 oxidation state, such as gadolinium, terbium, and dysprosium. By regarding the [N_2_]^2–^ ligand as a two-electron reservoir, compounds **1_M_** have potential to act as ‘synthons’ for [Cp_2_^ttt^M] in bespoke reduction chemistry, potentially across the full 4f series (except promethium). Proof of principle has been achieved with the synthesis of the radical-ligated complexes **2_M_**. Extension of the reactivity to, for example, tri-nucleating *N*-heterocycles would give access to strongly exchanged-coupled radical-bridged compounds, potentially with SMM properties. Furthermore, noting the recent demonstration of CO homologation by the isolable divalent thulium metallocene [Cp_2_^ttt^Tm],^24^ applications of **1_M_** in small-molecule activation with a broader range of lanthanides can be envisaged. This approach would complement existing methodologies involving stable divalent lanthanide metallocenes and sterically induced reduction.

## Conclusions

The targeted synthesis of metallocene-capped lanthanide dinitrogen complexes exclusively with the end-on μ-κ^1^:κ^1^-bridging mode has been achieved with **1_M_**. Formation of these compounds is aided by the bulk of the Cp^ttt^ ligand substituents in combination with the relatively small radii of gadolinium, terbium, and dysprosium. The high-yielding synthesis and stability at room temperature are noteworthy features of **1_M_**, as is the charge-neutral nature of the complexes, which eliminates considerations relating to alkali metal counter ions and associated chelate ligands. Raman spectroscopy revealed a major stretching vibration for each complex, located at 1623, 1621, and 1618 cm^–1^ for **1_Gd_**, **1_Tb_**, and **1_Dy_**, respectively, consistent with N=N double bonds in the [N_2_]^2–^ ligands. Analysis of the bonding in **1_Gd_** shows that the singlet form of the dinitrogen ligand is strongly preferred to the triplet form, which is a consequence of different spatial overlap between gadolinium 5d_π_ orbitals and the two dinitrogen π* orbitals.

DC magnetic susceptibility and magnetization measurements reveal that [N_2_]^2–^ mediates antiferromagnetic exchange between the Ln^3+^ ions, with AC susceptibility and magnetic hysteresis measurements also showing that **1_Dy_** is an SMM. A striking theoretical result from a study of **1_Tb_** and **1_Dy_** is the dominance of the dinitrogen ligand in the lanthanide crystal field, with the easy axis of magnetization being oriented along the {Ln–N=N–Ln} axis as opposed to aligning with the cyclopentadienyl ligands, as is commonly found.

By regarding [N_2_]^2–^ as a source of two electrons, compounds **1_M_** have been shown to react as synthons for divalent gadolinium, terbium, and dysprosium metallocenes. Proof-of-principle reactions led to the isolation of the radical-ligated complexes [Cp_2_^ttt^M(κ^2^-bipy)] (**2_M_**, M = Gd, Tb, Dy), which feature appreciable direct exchange coupling between the lanthanide and the bipy radical anion. SMM behavior was also found in **2_Dy_**. The electron-transfer reactivity of **1_M_** introduces possibilities for new directions in lanthanide small-molecule activation, the discovery of molecular magnets, and, potentially, for stoichiometric organic synthesis as alternatives to samarium(II) reductions.

## Data Availability

Additional research data supporting this publication are available as Supplementary Information at DOI: 10.25377/sussex.23703159.

## Supporting Information Available

Synthesis, spectroscopic characterization, crystallography details, magnetic measurements, computational details (PDF)
