# Supplementary material for: Targeted Synthesis of End-On Dinitrogen-Bridged Lanthanide Metallocenes and Their Reactivity as Divalent Synthons
Source: J Am Chem Soc. 2023 Sep 1;145(36):20121–31. doi: 10.1021/jacs.3c07600 (PMC10510326; doi:10.1021/jacs.3c07600)
Supplement: Supplementary file 1 — ja3c07600_si_001.pdf [file ja3c07600_si_001.pdf]

Targeted Synthesis of End-on Dinitrogen-bridged Lanthanide Metallocenes and their Reactivity as Divalent Synthons

Arpan Mondal,[a] Christopher G. T. Price,[a] Jinkui Tang,[b] Richard A Layfield*[a]

a Department of Chemistry, School of Life Sciences, University of Sussex, Brighton, BN1 9QR, U.K.
E- mail: r.layfield@sussex.ac.uk

b Changchun Institute of Applied Chemistry, Chinese Academy of Sciences, Renmin Street 5626, 130022 Changchun, P. R. China

Contents

Synthesis Details	S1-S3
X-Ray Crystallography	S3-S7
IR Spectra	S8-S9
EPR Spectroscopy	S10
DFT Calculations	S11-S12
Magnetic Property measurements	S13-S23
Ab Initio Calculations	S24-S30
References	S31

Synthesis Details

All reactions were carried out under rigorous anaerobic, anhydrous conditions using argon or nitrogen atmospheres and Schlenk and glove-box techniques. All solvents were refluxed over an appropriate drying agent for a minimum of three days (molten potassium for toluene and Na/K alloy for hexane), and then distilled and degassed via a minimum of three freeze-pump-thaw cycles. Solvents were then stored in ampoules over potassium mirrors. Elemental analyses were carried out at Elemental Microanalysis or MEDAC. Literature procedures were used to synthesise [Ln(BH_4)$_3$(THF)$_3$] (Ln = Gd, Tb, Dy),[1] Cp^{ttt}H,[2] and [(Cp^{ttt})$_2$Ln(BH_4)].[3] KCp^{ttt} was synthesised by reacting Cp^{ttt}H with KN($SiMe_3$)$_2$ in toluene overnight. FTIR spectra were recorded on a Bruker Alpha spectrometer with a platinum-diamond ATR module, housed in a glove box. Raman spectra were measured using a Renishaw inVia confocal Raman microscope using an excitation laser wavelength of 532 nm and 1800 mm^{-1} grating. Laser power was kept below 0.5 mW to reduce heating effects. Measurements used a 20× magnification objective, resulting in a laser spot size of about 1.60 μm. Spectra were baseline corrected and cosmic rays were removed using WiRE software. For the baseline, the software automatically uses an 'intelligent fitting', which excludes regions with peaks and fits the rest of the spectrum using a polynomial expression. UV-Visible spectra were recorded using a PerkinElmer LAMBDA 265 spectrophotometer. X-band EPR spectra were recorded in CW mode on a Bruker EMX spectrometer equipped with a Bruker ER049X SuperX microwave bridge, a Bruker ER4122SHQE resonator, and an Oxford Instruments ITC503 temperature controller.

Synthesis of [{(Cp^{ttt})$_2$Gd}$_2$(μ-1,2-N_2)] (1$_{Gd}$)

Hexane (20 mL) was added to a mixture of (Cp^{ttt})$_2$Gd(BH_4) (200 mg, 0.31 mmol) and KC_8 (216 mg, 1.57 mmol) at room temperature and the resulting suspension was stirred for seven days, during which time an intense orange coloured solution developed. The solution was filtered and the filtrate freeze-pump-thaw degassed, and then an excess of N_2 gas was admitted at 1 atmosphere pressure, which

resulted in the immediate formation of a deep blue suspension. The mixture was stored at –45°C overnight to effect complete precipitation of the product, which was isolated by filtration and dried under vacuum to yield $\mathbf{1_{Gd}}$ as a brown crystalline solid (175 mg, 87% based on Gd).

Elemental analysis (%), found (calculated) for $C_{68}H_{116}Gd_2N_2$: C 64.15 (64.00); H 8.99 (9.16); N 2.10 (2.20). **FTIR** ($\tilde{\nu}$/cm^{-1}): 3000-2800 (m, br, C-H). **Raman** ($\tilde{\nu}$/cm^{-1}): 1623 cm^{-1} (N_2 stretch).

Synthesis of [{(Cp^{ttt})$_2$Tb}$_2$(μ-1,2-N$_2$)] ($\mathbf{1_{Tb}}$)

Compound $\mathbf{1_{Tb}}$ was synthesised using the procedure described for $\mathbf{1_{Gd}}$, using (Cp^{ttt})$_2$Tb(BH$_4$) (200 mg, 0.30 mmol) and KC_8 (201 mg, 1.50 mmol), and isolated as a brown crystalline solid (159 mg, 83% based on Tb).

Elemental analysis (%), found (calculated) for $C_{68}H_{116}Tb_2N_2$: C 63.77 (63.83); H 9.11 (9.14); N 2.11 (2.19). **FTIR** ($\tilde{\nu}$/cm^{-1}): 3000-2800 (m, br, C-H). **Raman** ($\tilde{\nu}$/cm^{-1}): 1621 cm^{-1} (N_2 stretch).

Synthesis of [{(Cp^{ttt})$_2$Dy}$_2$((μ-1,2-N$_2$)] ($\mathbf{1_{Dy}}$)

Compound $\mathbf{1_{Dy}}$ was synthesised via the same procedure as for $\mathbf{1_{Gd}}$, using (Cp^{ttt})$_2$Dy(BH$_4$) (200 mg, 0.30 mmol) and KC_8 (200 mg, 1.50 mmol), and isolated as a blue crystalline solid (160 mg, 84% based on Dy).

Elemental analysis (%), found (calculated) for $C_{68}H_{116}Dy_2N_2$: C 63.38 (63.48); H 9.14 (9.09); N 2.10 (2.18). **FTIR** ($\tilde{\nu}$/cm^{-1}): 3000-2800 (m, br, C-H). **Raman** ($\tilde{\nu}$/cm^{-1}): 1618 cm^{-1} (N_2 stretch).

Solution stability of $\mathbf{1_M}$

Compounds $\mathbf{1_M}$ have poor solubility. Suspensions of $\mathbf{1_M}$ decompose to unknown material within 24 hours at room temperature (checked with pentane, hexane, benzene, toluene, chlorobenzene, Et_2O and $(Me_3Si)_2O$ and THF). Solid samples appear stable indefinitely in the glovebox at room temperature.

Synthesis of [(Cp^{ttt})$_2$Gd(bipy)] ($\mathbf{2_{Gd}}$)

Toluene (10 mL) was added to a mixture of $\mathbf{1_{Gd}}$ (100 mg, 0.08 mmol) and 2,2'-bipyridine (24.4 mg, 0.16 mmol) and the suspension was stirred at room temperature for 16 hours, producing a deep green solution. The solvent was then removed in vacuo to give a solid residue, which was redissolved in the minimum amount of hexane (8 mL). Storing the solution at –45 °C for three days led to the formation of deep green crystals of $\mathbf{2_{Gd}}$ (55 mg, 90%).

Elemental analysis (%), found (calculated) for $C_{44}H_{66}GdN_2$: C 67.69 (67.73); H 8.49 (8.53); N 3.55 (3.59). **FTIR** ($\tilde{\nu}$/cm^{-1}): 3000-2800 (m, br, C-H).

Synthesis of [(Cp^{ttt})$_2$Tb(bipy)] ($\mathbf{2_{Tb}}$)

Compound $\mathbf{2_{Tb}}$ was synthesised via the same procedure as for $\mathbf{2_{Gd}}$, using $\mathbf{1_{Tb}}$ (100 mg, 0.08 mmol) and 2,2'-bipyridine (24.4 mg, 0.16 mmol), and isolated as a green crystalline solid (58 mg, 95%).

Elemental analysis (%), found (calculated) for $C_{44}H_{66}TbN_2$: C 67.41 (67.59); H 8.45 (8.51); N 3.51 (3.58). **FTIR** ($\tilde{\nu}$/cm^{-1}): 3000-2800 (m, br, C-H).

Synthesis of [(Cp^{ttt})$_2$Dy(bipy)] ($\mathbf{2_{Dy}}$)

Compound **2_{Dy}** was synthesised via the same procedure as for **2_{Gd}**, using **1_{Dy}** (100 mg, 0.08 mmol) and 2,2'-bipyridine (24.3 mg, 0.16 mmol), and isolated as green crystalline solid (50 mg, 82%).

Elemental analysis (%), found (calculated) for $C_{44}H_{66}DyN_2$: C 67.18 (67.28); H 8.46 (8.47); N 3.49 (3.57). **FTIR** ($\tilde{\upsilon}$/cm^{-1}): 3000-2800 (m, br, C-H).

X-ray Crystallography

Single-crystal X-ray diffraction measurements were carried out on an Agilent Gemini Ultra or a Rigaku HyPix 6000HE diffractometer using Cu-K$_\alpha$ radiation (λ = 1.54184 Å) at 100 K. Crystals were mounted on MiTiGen loops from pump oil kept over activated 4 Å molecular sieves in a glove box. Data collection and processing was handled by CrysAlis Pro. Structure solution and model refinement were performed using the Olex2 package and all software within.[4,5] Anisotropic thermal parameters were used for non-hydrogen atoms and isotropic parameters for hydrogen atoms. Hydrogen atoms on carbons were added geometrically and refined using a riding model. Solvent masking was used in the refinement of **1_{Gd}**, **1_{Tb}**, and **1_{Dy}** due to the presence of highly disordered pentane molecules in the lattice.

Table S1. Crystal data and structure refinement parameters for **1_{Gd}**, **1_{Tb}**, and **1_{Dy}**.

	**1_{Gd}**	**1_{Tb}**	**1_{Dy}**
CCDC ref. code	2267073	2267075	2267074
Empirical formula	$C_{68}H_{116}Gd_2N_2$	$C_{68}H_{116}Tb_2N_2$	$C_{68}H_{116}Dy_2N_2$
Formula weight	1276.12	1279.46	1286.62
Crystal system	Monoclinic	Monoclinic	Monoclinic
Space group	*P*2/*n*	*P*2/*n*	*P*2/*n*
a (Å)	18.5187(7)	18.5412(9)	18.4948(8)
b (Å)	10.3368(3)	10.3359(4)	10.3501(4)
c (Å)	19.7354(8)	19.7615(8)	19.4793(9)
α (°)	90	90	90
β (°)	110.421(4)	110.475(5)	110.162(5)
γ (°)	90	90	90
V (Å^3)	3540.4(2)	3547.8(3)	3500.3(3)
Z	2	2	2
ρ_{calc} (g cm^{-3})	1.197	1.198	1.221
F (000)	1332.0	1336.0	1340.0
Reflections collected	36802	45554	42239
Independent reflections	6698	6296	6223
R_{int} (%)	0.0478	0.1021	0.1354
GOF on F^2	1.089	1.1011	1.115
R_1[a]	0.0465	0.0511	0.0691
wR_2[b]	0.1344	0.1411	0.2115

[a] $R_1[I>2\sigma(I)] = \sum||F_o|-|F_c||/\sum|F_o|$; [b] wR_2[all data] = $[\sum\{w(F_o^2-F_c^2)^2\}/\sum\{w(F_o^2)^2\}]^{1/2}$

Table S2. Crystal data and structure refinement parameters for $\mathbf{2_{Gd}}$, $\mathbf{2_{Tb}}$, and $\mathbf{2_{Dy}}$.

	$\mathbf{2_{Gd}}$	$\mathbf{2_{Tb}}$	$\mathbf{2_{Tb}}$
CCDC ref. code	2267070	2267071	2267072
Empirical formula	$C_{44}H_{66}GdN_2$	$C_{44}H_{66}TbN_2$	$C_{44}H_{66}DyN_2$
Formula weight	780.23	781.90	785.48
Crystal system	Monoclinic	Monoclinic	Monoclinic
Space group	*C*2/*c*	*C*2/*c*	*C*2/*c*
a (Å)	13.33690(10)	13.32110(10)	13.2963(2)
b (Å)	16.92840(10)	17.0408(2)	17.0467(2)
c (Å)	17.7224(2)	17.6325(2)	17.6135(2)
α (°)	90	90	90
β (°)	97.1960(10)	97.2460(10)	97.1310(10)
γ (°)	90	90	90
V (Å^3)	3969.71(6)	3970.65(7)	3961.36(9)
Z	4	4	4
ρ_{calc} (g cm^{-3})	1.305	1.308	1.317
F (000)	1632.0	1636.0	1640.0
Reflections collected	18776	26327	35040
Independent reflections	3505	3511	3494
R_{int} (%)	0.0303	0.0815	0.0566
GOF on F^2	1.042	1.090	1.047
R_1[a]	0.0226	0.0342	0.0265
wR_2[b]	0.0586	0.0865	0.0687

[a] $R_1[I>2\sigma(I)] = \sum||F_o|-|F_c||/$[illegible]$|F_o|$; [b] wR_2[all data] $= [\sum\{w(F_o^2-F_c^2)^2\}/\sum\{w(F_o^2)^2\}]^{1/2}$

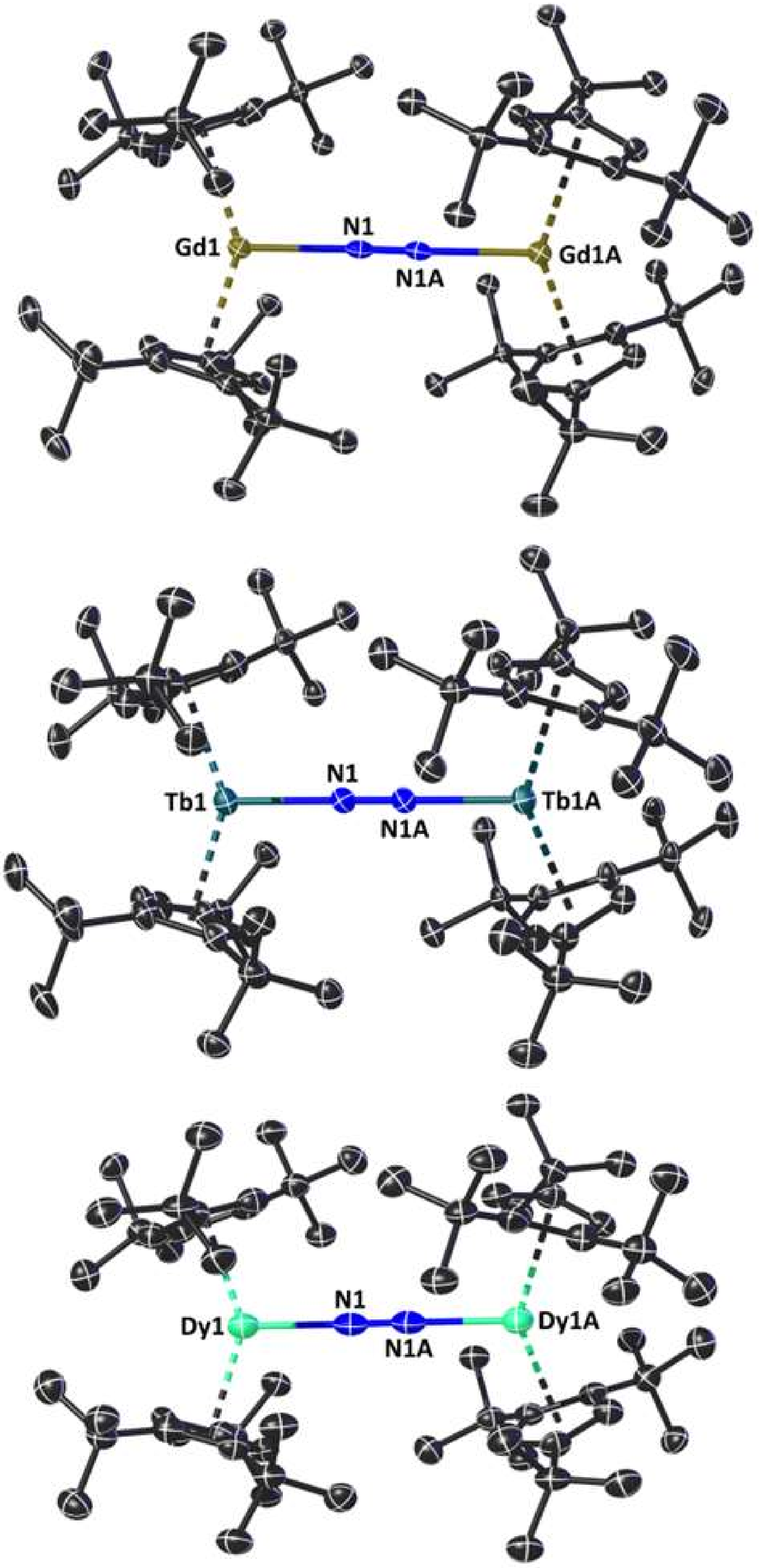

Figure S1. Thermal ellipsoid representations (30% probability) of $\mathbf{1}_{\mathbf{Gd}}$ (top), $\mathbf{1}_{\mathbf{Tb}}$ (middle) and $\mathbf{1}_{\mathbf{Dy}}$ (bottom). Hydrogen atoms are omitted for clarity.

Table S3. Selected bond lengths (Å) and angles (°) for $\mathbf{1}_{Gd}$, $\mathbf{1}_{Tb}$, and $\mathbf{1}_{Dy}$.

	$\mathbf{1}_{Gd}$	$\mathbf{1}_{Tb}$	$\mathbf{1}_{Dy}$
M–C	Gd1-C1: 2.690(4)<br>Gd1-C2: 2.763(4)<br>Gd1-C3: 2.757(4)<br>Gd1-C4: 2.694(4)<br>Gd1-C5: 2.715(4)<br>Gd1-C18: 2.712(4)<br>Gd1-C19: 2.700(4)<br>Gd1-C20: 2.687(4)<br>Gd1-C21: 2.758(4)<br>Gd1-C22: 2.753(4)	Tb1-C1: 2.738(4)<br>Tb1-C2: 2.741(4)<br>Tb1-C3: 2.662(5)<br>Tb1-C4: 2.700(4)<br>Tb1-C5: 2.668(5)<br>Tb1-C18: 2.673(5)<br>Tb1-C19: 2.734(4)<br>Tb1-C20: 2.740(4)<br>Tb1-C21: 2.670(5)<br>Tb1-C22:2.706(5)	Dy1-C1: 2.686(6)<br>Dy1-C2: 2.661(7)<br>Dy1-C3: 2.736(7)<br>Dy1-C4: 2.718(6)<br>Dy1-C5: 2.652(7)<br>Dy1-C6: 2.726(6)<br>Dy1-C7: 2.646(7)<br>Dy1-C8: 2.693(6)<br>Dy1-C9: 2.672(8)<br>Dy1-C10: 2.731(6)
M–Cp^{ttt}_{cent}	2.4370(16)<br>2.4399(17)	2.4146(19)<br>2.419(2)	2.405(3)<br>2.406(3)
M1–N1	2.325(4)	2.296(4)	2.268(7)
N1–N1A	1.130(8)	1.175(8)	1.215(13)
M1···M1A	5.7791(9)	5.7659(9)	5.7500(9)
Cp^{ttt}_{cent}–M–Cp^{ttt}_{cent}	144.63(8)	143.96(9)	143.86(13)

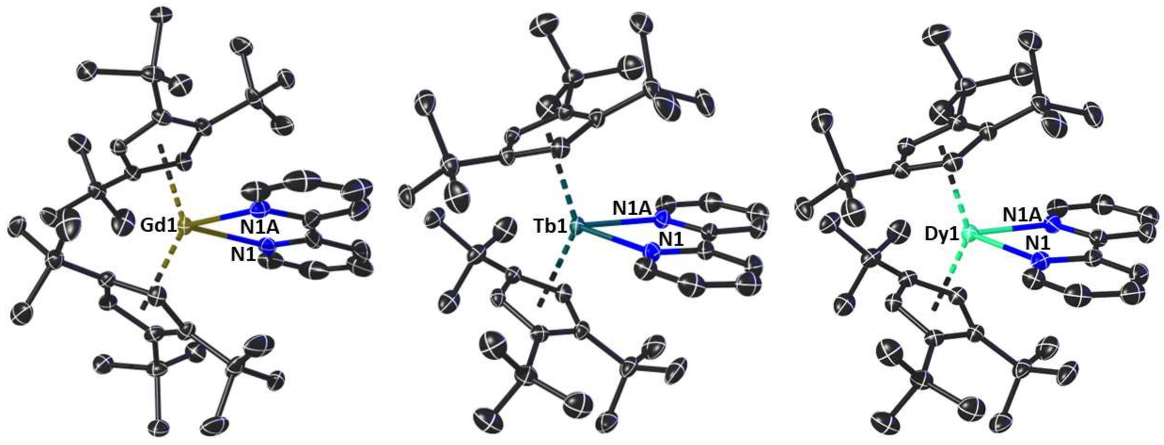

Figure S2. Thermal ellipsoid representations (50% probability) of **2**$_{Gd}$ (left), **2**$_{Tb}$ (middle) and **2**$_{Dy}$ (right). Hydrogen atoms are omitted for clarity.

Table S4. Selected bond lengths (Å) and angles (°) for **2**$_{Gd}$, **2**$_{Tb}$ and **2**$_{Dy}$.

	**2**$_{Gd}$	**2**$_{Tb}$	**2**$_{Dy}$
M–C	Gd1-C1: 2.826(2)<br>Gd1-C2: 2.726(2)<br>Gd1-C3: 2.750(2)<br>Gd1-C4: 2.750(2)<br>Gd1-C5: 2.846(2)	Tb1-C1: 2.699(3)<br>Tb1-C2: 2.739(3)<br>Tb1-C3: 2.739(3)<br>Tb1-C4: 2.836(3)<br>Tb1-C5: 2.794(3)	Dy1-C1: 2.724(2)<br>Dy1-C2: 2.821(2)<br>Dy1-C3: 2.778(3)<br>Dy1-C4: 2.679(2)<br>Dy1-C5: 2.724(2)
M–Cp^{ttt}_{cent}	2.5036(10)	2.4832(14)	2.4650(11)
M1–N1	2.4052(18)	2.384(3)	2.365(2)
Cp^{ttt}_{cent}–M–Cp^{ttt}_{cent}	140.17(5)	141.04(7)	141.28(5)

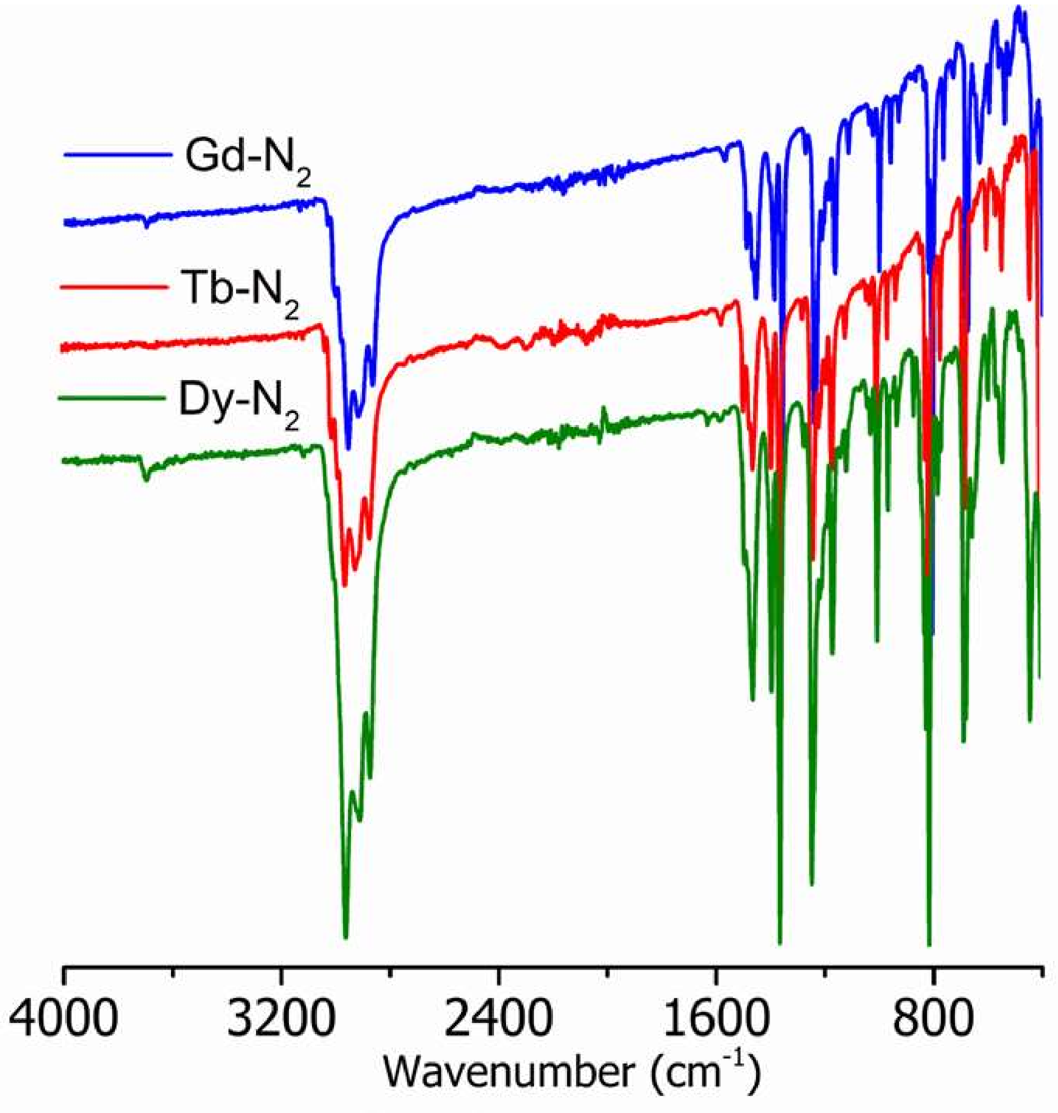

Figure S3. FTIR spectra of $\mathbf{1}_{\mathbf{Gd}}$, $\mathbf{1}_{\mathbf{Tb}}$, and $\mathbf{1}_{\mathbf{Dy}}$.

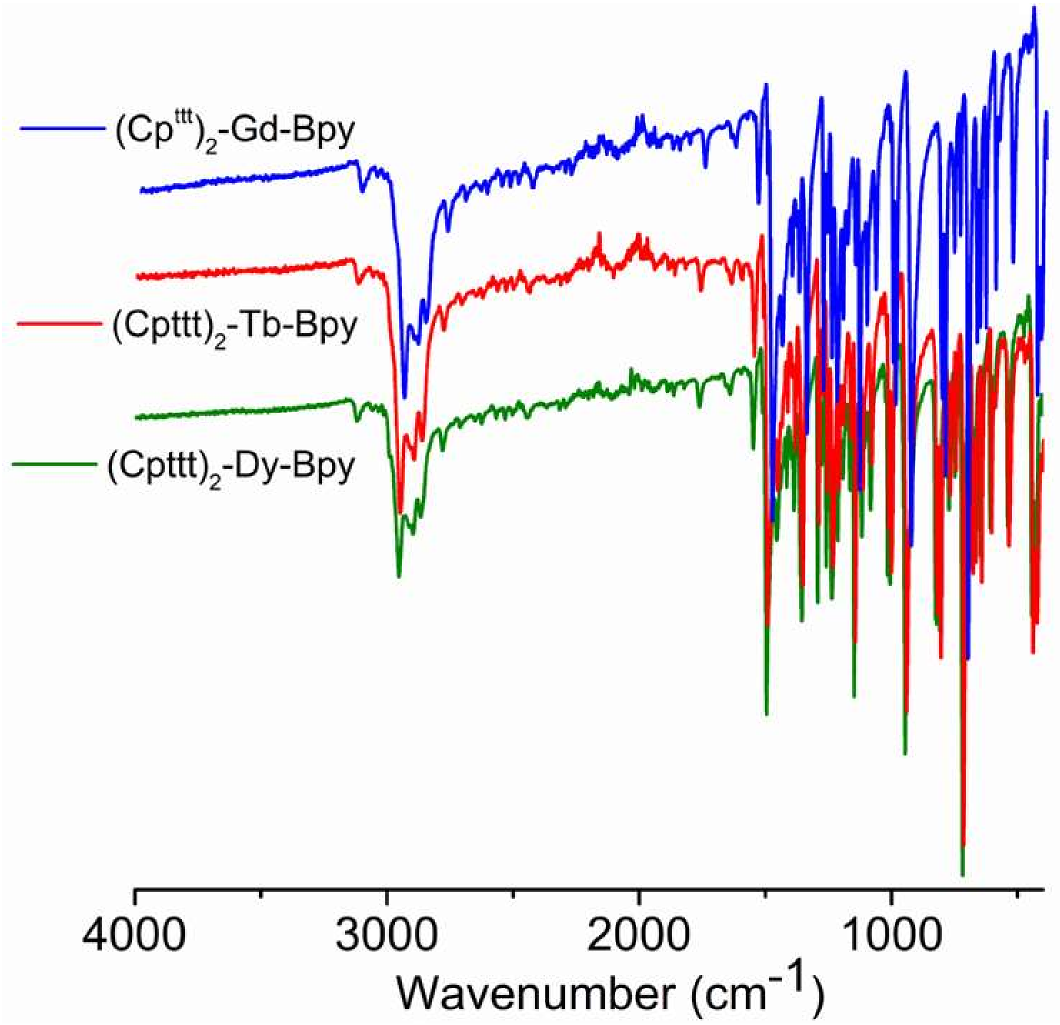

Figure S4. FTIR spectra of $\mathbf{2_{Gd}}$, $\mathbf{2_{Tb}}$, and $\mathbf{2_{Dy}}$.

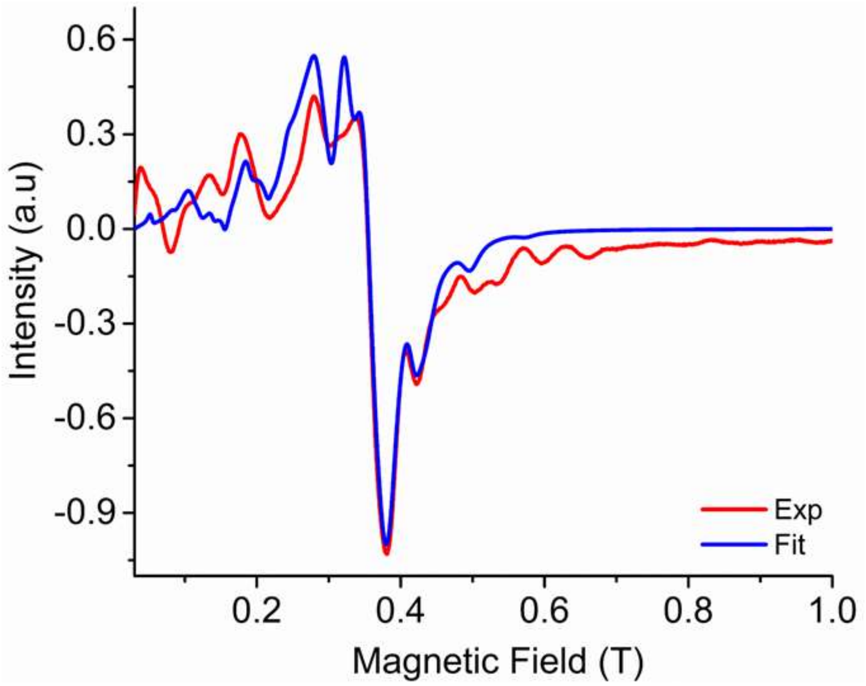

Figure S5. X-Band EPR spectrum of $\mathbf{1}_{\mathbf{Gd}}$ in the solid-state at 298 K. Fitting of the spectrum was achieved using $H = g\mu_{\mathrm{B}}SB + D[S_z^2 - S(S+1)/3] + E(S_x^2 - S_y^2)$ with $g = 2.001$, $D = -0.014\ \mathrm{cm}^{-1}$ and $E = -0.0013\ \mathrm{cm}^{-1}$, giving a rhombicity parameter of $E/D = 0.09$.

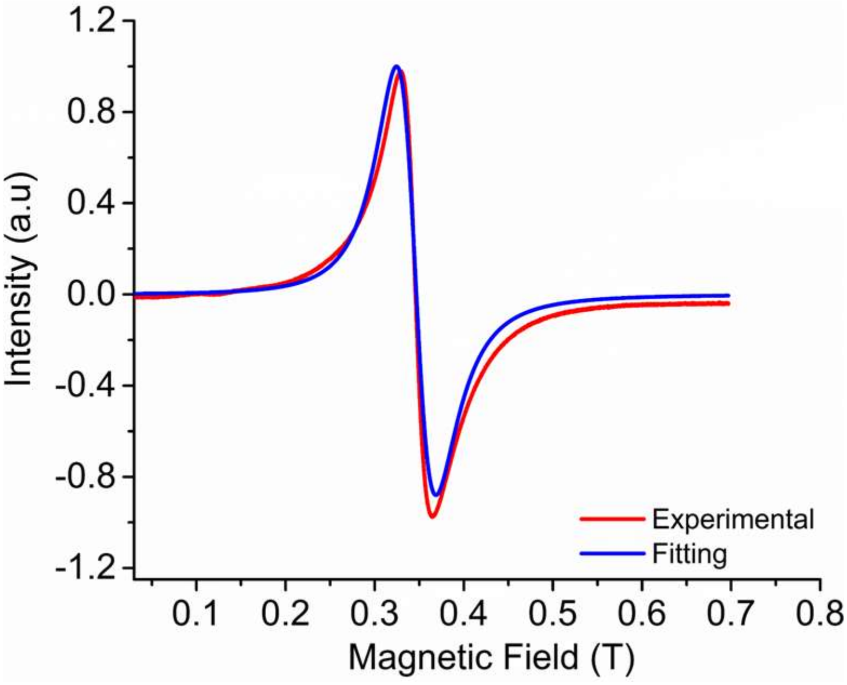

Figure S6. X-Band EPR spectrum of $\mathbf{2}_{\mathbf{Gd}}$ in the solid-state at 298 K. Fitting of the spectrum was achieved using $H = g\mu_{\mathrm{B}}SB + D[S_z^2 - S(S+1)/3] + E(S_x^2 - S_y^2)$ with $g = 1.98$, $D = -0.011\ \mathrm{cm}^{-1}$ and $E = -0.0010\ \mathrm{cm}^{-1}$, giving a rhombicity parameter of $E/D = 0.09$.

DFT calculations

The DFT calculations on $\mathbf{1}_{Gd}$ were performed on coordinates obtained from the X-ray structure using Gaussian09 programme without optimizations, except for the hydrogen atoms. The hybrid B3LYP functional[6] was used along with the effective core potential 'Stuttgart RSC 1997' basis set[7] for gadolinium, and the Ahlrichs triple-ζ TZV basis set[8] for other atoms. The NBO analysis was performed using NBO 3.0 as implemented in Gaussian09. The TD-DFT calculations were performed with the PBE0 hybrid functional[9] using ORCA 5.0.2.[10] The relativistic effects were included with the Douglas-Kroll-Hess (DKH) Hamiltonian, together with the scalar relativistic contracted version of the basis functions def2-QZVP for Gd, and def2-TZVP for N and def2-SVP for other atoms. The CPCM solvation model was used to consider solvent effects.

To gain insight into the magnetic exchange coupling, we also performed DFT calculations in combination with the broken symmetry (BS) approach in ORCA. Relativistic effects were included with the DKH Hamiltonian, together with the scalar relativistic contracted version of the basis functions def2-QZVP for gadolinium and def2-TZVP for other atoms. In these calculations, the well-known B3LYP functional was employed to extract the isotropic exchange coupling constant (J) using equation S1, where E_{T}, E_{BS} and S represent the energy of the triplet state, broken-symmetry state and total spin, respectively.

$$J = \frac{-(E_{\mathrm{T}} - E_{\mathrm{BS}})}{S(S+1)} \quad \text{(S1)}$$

For $\mathbf{1}_{Gd}$, the energy of the high-spin (triplet) state was determined as $E_{\mathrm{T}} = –25300.704939$ hartree and the broken symmetry state as $E_{\mathrm{BS}} = –25300.707329$ hartree, leading to $J = –9.37$ cm$^{–1}$.

For $\mathbf{2}_{Gd}$, the energy of the high-spin (triplet) state was determined as $E_{\mathrm{T}} = –13349.623384$ hartree and the broken symmetry state as $E_{\mathrm{BS}} = –13349.624128$ hartree, leading to $J = –8.16$ cm$^{–1}$.

Table S5. DFT calculated spin densities on selected atoms for $\mathbf{1}_{Gd}$.

**Atom**	**Spin**
Gd1	7.075
N1	-0.071
Gd2	7.075
N2	-0.071

Table S6. Selected bonding orbitals from the natural bond orbital (NBO) analysis of $\mathbf{1}_{Gd}$.

**(Occupancy) Bond**	**Composition**
(0.82709) BD (1) Gd1 - N2	(14.09%) 0.3753*Gd 1 s(0.00%)p 1.00(9.52%)d 9.47(90.17%)f 0.03(0.30%)<br>(85.91%) 0.9269* N 2 s(0.00%)p 1.00(99.96%)d 0.00(0.04%)
(0.99700) BD (1) N2 - N95	(50.00%) 0.7071* N 2 s(0.00%)p 1.00(99.77%)d 0.00(0.23%)<br>(50.00%) 0.7071* N 95 s(0.00%)p 1.00(99.77%)d 0.00(0.23%)
(0.99485) BD (2) N2 - N95	(50.00%) 0.7071* N 2 s(42.99%)p 1.32(56.83%)d 0.00(0.18%)<br>(50.00%) 0.7071* N 95 s(42.99%)p 1.3256.83%)d 0.00(0.18%)

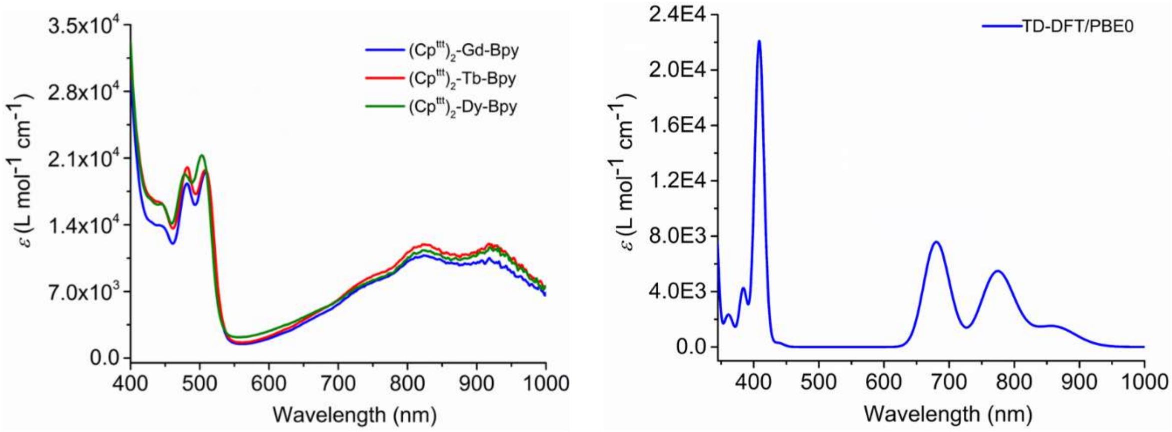

Figure S7. Left: experimental UV-vis absorbance spectra for $\mathbf{2_M}$ in hexane. Right: TD-DFT calculated UV-vis absorbance spectrum for $\mathbf{2_{Gd}}$ in hexane.

Table S7. Computed excitation wavelengths (λ) and oscillator strengths (f) in length representation for $\mathbf{2_{Gd}}$.

**Excitation**	**λ / nm**	***f***	**Assignment**
207a → 209a	861.3	0.006	$[Bipy]^-$ π*→π*
207a → 208a	774.5	0.025	$[Bipy]^-$ π*→d(Gd)
207a → 210a	680.3	0.034	$[Bipy]^-$ π*→π*
207a → 211a	437.7	0.001	$[Bipy]^-$ π*→d(Gd)
207a → 212a	408.5	0.101	$[Bipy]^-$ π*→π*
199b → 200b	384.0	0.019	$[Bipy]^-$ π→π*
198b → 200b	366.1	0.006	$[Bipy]^-$ π→π*

Magnetic measurements

Magnetic measurements were recorded on a Quantum Design MPMS-XL7 SQUID magnetometer equipped with a 7 T (70 kOe) magnet. The samples were restrained in eicosane and sealed in 7 mm NMR tubes. Direct current (DC) magnetic susceptibility measurements were performed on polycrystalline samples in the temperature range 1.9-300 K and using an applied field of 1000 Oe. Alternating current (AC) susceptibility measurements were performed using an AC field of 3 Oe in zero DC field. Diamagnetic corrections were made using Pascal's constants for all the constituent atoms.[11]

Simulation of Magnetic Susceptibility and Magnetization data

Simulations for $[\{(Cp^{ttt})_2M\}_2(\mu\text{-}1,2\text{-}N_2)]$ ($\mathbf{1_M}$, M = Gd, Tb, Dy)

To quantify the exchange interactions, we have simulated the molar magnetic susceptibility and magnetization data for all complexes using the PHI software.[9] For $\mathbf{1_{Gd}}$, the isotropic Hamiltonian stated as equation 1 in the main text was used, whereas the Hamiltonian stated as equation 2 in the main text was used for $\mathbf{1_{Tb}}$ and $\mathbf{1_{Dy}}$.

$$\hat{H} = -2J(S_{\mathrm{Gd1}} \cdot S_{\mathrm{Gd2}}) + \beta(g_{\mathrm{Gd1}} \cdot S_{\mathrm{Gd1}} + g_{\mathrm{Gd2}} \cdot S_{\mathrm{Gd2}}) \cdot B \quad (1)$$

$$\hat{H} = -2J(S_{\mathrm{Ln1}} \cdot S_{\mathrm{Ln2}}) + \beta(g_{\mathrm{Ln1}} \cdot S_{\mathrm{Ln1}} + g_{\mathrm{Ln2}} \cdot S_{\mathrm{Ln2}}) \cdot B + B_2^0 C_2^0 + B_4^0 C_4^0 + B_6^0 C_6^0 \quad (2)$$

For $\mathbf{1_{Tb}}$ and $\mathbf{1_{Dy}}$, we considered the crystal field parameters obtained from the ab initio calculations (Table S11) and an intermolecular interaction term, zJ, calculated to be +0.01 cm^{-1} and –0.05 cm^{-1}, respectively. The best simulation of both susceptibility and magnetization results in weak antiferromagnetic interactions for all complexes.

Simulations for $[(Cp^{ttt})_2M(bipy)]$ ($\mathbf{2_M}$, M = Gd, Tb, Dy)

To quantify the exchange interaction between gadolinium and the bipy radical anion ligand in $\mathbf{2_{Gd}}$, we performed a simulation using an isotropic Hamiltonian with an intermolecular term $zJ = -0.15$ cm^{-1}, as expressed in equation 4 in the main text. The Hamiltonian stated as equation 5 in the main text, which includes the ab initio crystal field parameters, was used for $\mathbf{2_{Tb}}$ and $\mathbf{2_{Dy}}$.

$$\hat{H} = -2J(S_{\mathrm{rad}} \cdot S_{\mathrm{Gd}}) + \beta(g_{\mathrm{Gd}} \cdot S_{\mathrm{Gd}} + g_{\mathrm{rad}} \cdot S_{\mathrm{rad}}) \cdot B \quad (4)$$

$$\hat{H} = -2J(S_{\mathrm{rad}} \cdot S_{\mathrm{Ln}}) + \beta(g_{\mathrm{Ln}} \cdot S_{\mathrm{Ln}} + g_{\mathrm{rad}} \cdot S_{\mathrm{rad}}) \cdot B + B_2^0 C_2^0 + B_4^0 C_4^0 + B_6^0 C_6^0 \quad (5)$$

For $\mathbf{2_{Tb}}$ and $\mathbf{2_{Dy}}$, the crystal field parameters were calculated for the hypothetical oxidized complexes $[(Cp^{ttt})_2Tb(bipy)]^+$ and $[(Cp^{ttt})_2Dy(bipy)]^+$ in order to simplify the active space (Table S15). Intermolecular terms of $zJ = -0.45$ cm^{-1} and $zJ = -0.60$ cm^{-1}, respectively, were used in the simulations. For all $\mathbf{2_M}$ complexes, reasonable simulations of the $\chi_M T(T)$ data were obtained, but the $M(H)$ data could not accurately be reproduced.

Table S8. Parameters used to simulate the magnetic data.

**Complex**	***J* / cm^{-1}**	***g***	**CF parameter / cm^{-1}**
**1$_{Gd}$**	–0.81	1.9	
**1$_{Tb}$**	–0.65	1.5 (g_J)	B_2^0 = –6.520<br>B_4^0 = 0.00809<br>B_6^0 = 1.45 × 10^{-5}
**1$_{Dy}$**	–0.07	1.33 (g_J)	B_2^0 = –3.64<br>B_4^0 = –0.002420<br>B_6^0 = 4.75 × 10^{-6}
**2$_{Gd}$**	–5.50	$g_{\mathrm{rad}} = 2.0$<br>$g_{\mathrm{Gd}} = 2.0$	
**2$_{Tb}$**	–4.65	$g_{\mathrm{rad}} = 2.0$<br>$g_{\mathrm{Tb}} = 1.51$	B_2^0 = –4.79<br>B_4^0 = –0.00622<br>B_6^0 = 1.76 × 10^{-5}
**2$_{Dy}$**	–3.50	$g_{\mathrm{rad}} = 2.0$<br>$g_{\mathrm{Dy}} = 1.36$	B_2^0 = –2.45<br>B_4^0 = –0.001619<br>B_6^0 = 1.616 × 10^{-5}

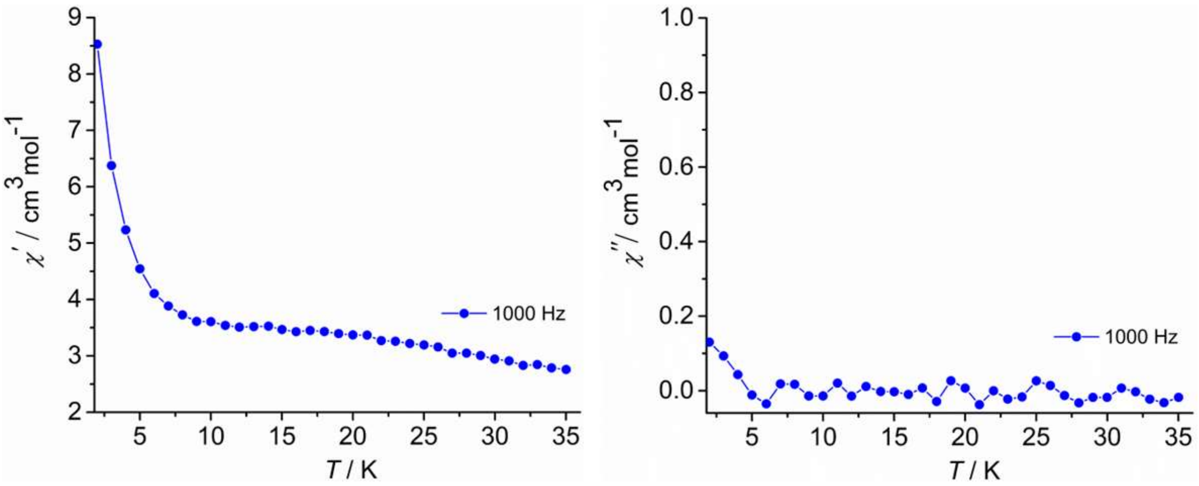

Figure S8. Real (left) and imaginary (right) components of the AC susceptibility as a function of temperature at 1000 Hz frequency in an AC field of 3 Oe and zero DC field for $\mathbf{1}_{\mathbf{Tb}}$.

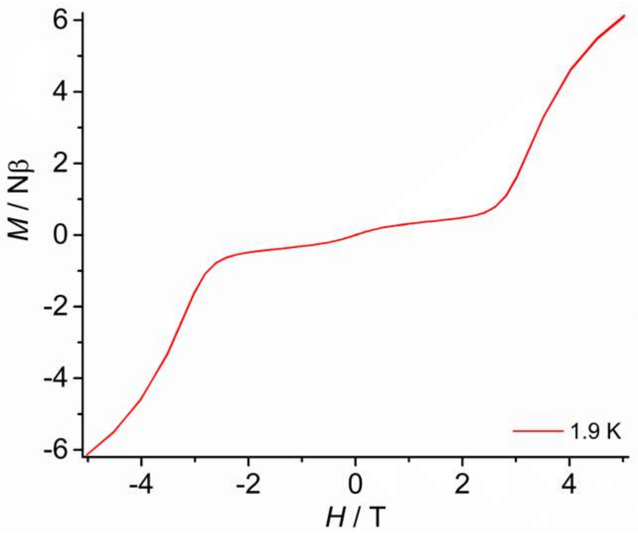

Figure S9. Magnetic hysteresis plot for $\mathbf{1}_{\mathbf{Tb}}$. The data were continuously collected at 1.9 K under the following field sweep rates: 5 mT s^{-1} |0-0.02| T; 10 mT s^{-1} |0.02-0.1| T; 50 mT s^{-1} |0.1-0.40| T; 100 mT s^{-1} |0.4-2.0| T; 200 mT s^{-1} |2.0-3.0| T; 500 mT s^{-1} |3.0-5.0| T.

S1

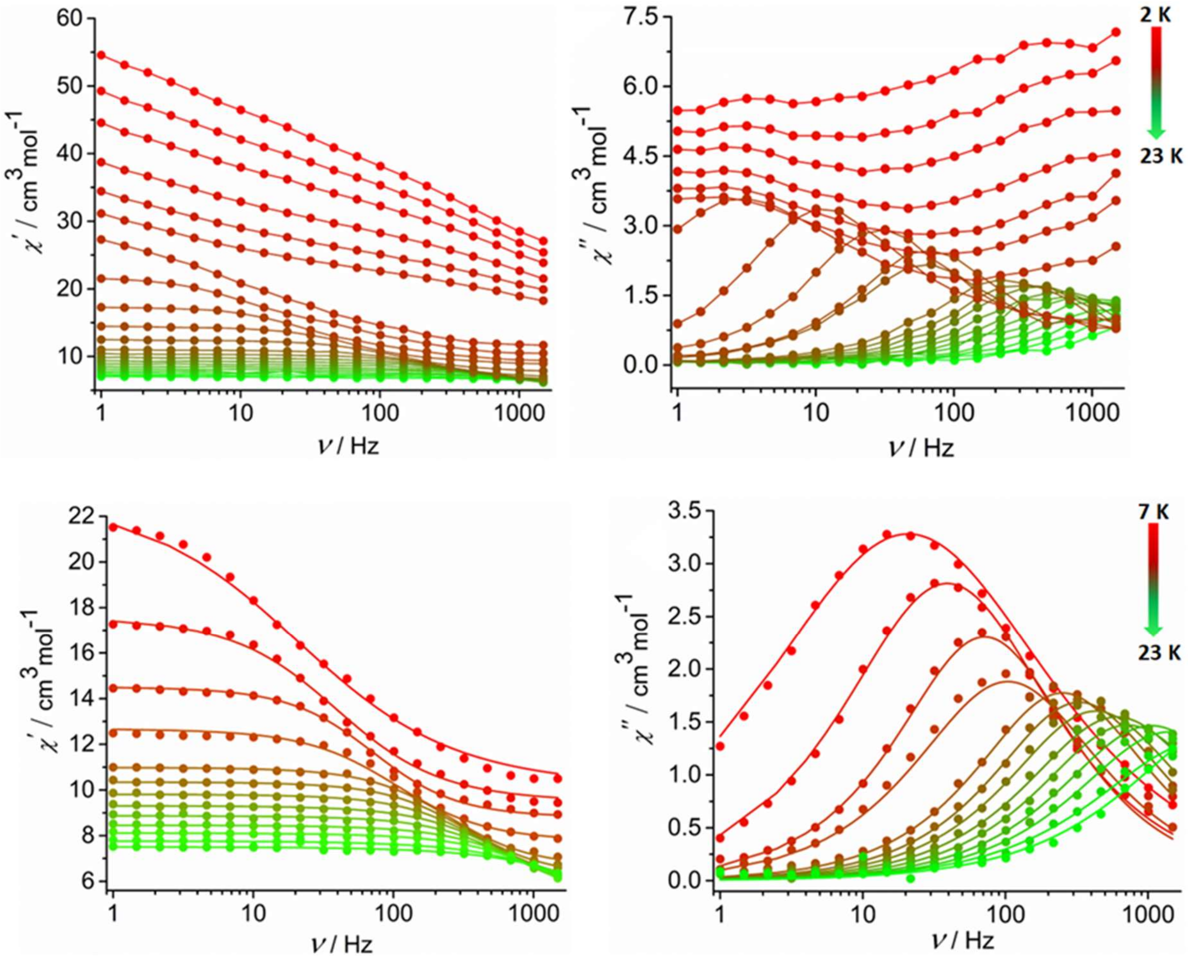

Figure S10. Upper: real (left) and imaginary (right) components of the AC susceptibility as a function of frequency at T = 2-23 K in an AC field of 3 Oe and zero DC field for $\mathbf{1}_{\mathbf{Dy}}$. Lower: the same AC data in the temperature range T = 7-23 K.

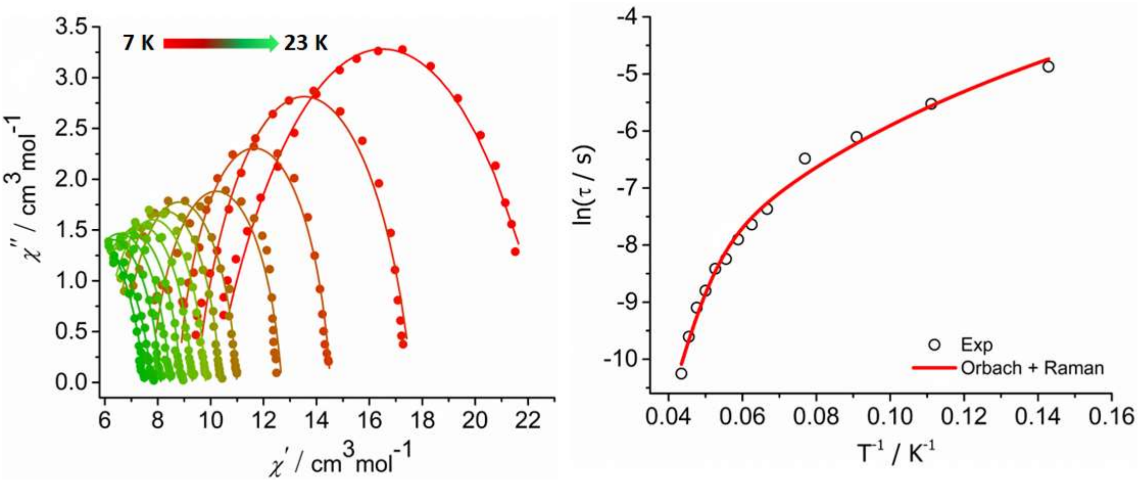

Figure S11. Cole-Cole plot (left) and temperature-dependence of the relaxation time (right) for $\mathbf{1}_{\mathbf{Dy}}$, where solid lines are fits to the data according to equations S2 and S3 and the parameters in Table S9.

$$\chi'(\nu) = \chi_S + \frac{(\chi_T - \chi_S)[1 + (2\pi\nu\ )^{(1-\alpha)}\ sin(\frac{\alpha\pi}{2})]}{1 + 2(2\pi\nu\tau)^{(1-\alpha)}\ sin\left(\frac{\alpha\pi}{2}\right) + (2\pi\nu\tau)^{2(1-\alpha)}} \text{....(S2)}$$

$$\chi''(\nu) = \frac{(\chi_T - \chi_S)(2\pi\nu\tau)^{(1-\alpha)}\ cos(\frac{\alpha\pi}{2})}{1 + 2(2\pi\nu\tau)^{(1-\alpha)}\ sin\left(\frac{\alpha\pi}{2}\right) + (2\pi\nu\tau)^{2(1-\alpha)}} \text{....(S3)}$$

Table S9. Relaxation fitting parameters for $\mathbf{1_{Dy}}$ for the Cole-Cole plot in Figure S11.

**T / K**	**χ_S / cm^3 mol^{-1}**	**χ_T / cm^3 mol^{-1}**	**τ / s**	**α**
7	10.13604	22.88165	0.00765	0.39459
9	9.47017	17.56708	0.00399	0.21951
11	8.78124	14.52218	0.00223	0.13852
13	7.74547	12.68475	0.00153	0.17106
15	6.62228	10.98845	6.31624E-4	0.13099
16	6.22728	10.35111	4.81338E-4	0.12715
17	5.83119	9.81178	3.70679E-4	0.13678
18	5.36358	9.30853	2.6326E-4	0.15322
19	5.18289	8.87014	2.21623E-4	0.14259
20	4.67731	8.45878	1.50895E-4	0.16056
21	4.38701	8.11292	1.12155E-4	0.1765
22	3.91911	7.76742	6.73512E-5	0.23752
23	2.80154	7.50931	3.52933E-5	0.25425

Fitting of the temperature-dependence of the relaxation times for $\mathbf{1_{Dy}}$ was achieved with the following parameters:

$$\tau^{-1} = \tau_0^{-1} exp^{(-\frac{U_{eff}}{k_B T})} + CT^n$$

C	0.20 ± 0.05 s^{-1} K^{-n}
n	3.27 ± 0.25
U_{eff}	180 ± 37 cm^{-1}
τ_0	7.30 × 10^{-10} s

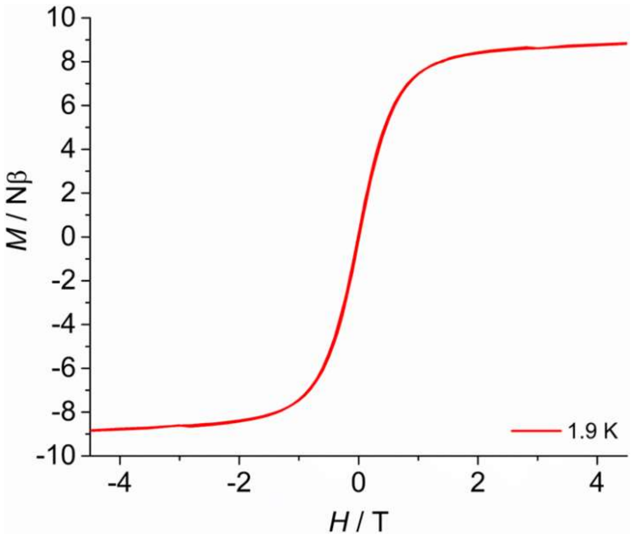

Figure S12. Magnetic hysteresis plot for $\mathbf{1_{Dy}}$. The data were continuously collected at 1.9 K under the following field sweep rates: 5 mT s^{-1} |0-0.02| T; 10 mT s^{-1} |0.02-0.1| T; 50 mT s^{-1} |0.1-0.40| T; 100 mT s^{-1} |0.4-2.0| T; 200 mT s^{-1} |2.0-3.0| T; 500 mT s^{-1} |3.0-5.0| T.

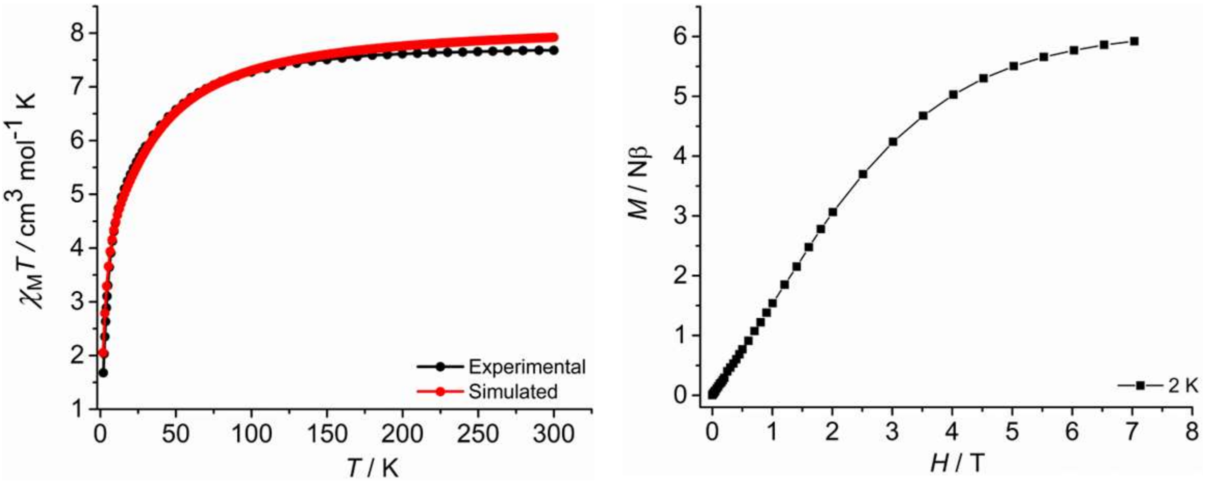

Figure S13. Left: $\chi_{\mathrm{M}}T(T)$ for **2**$_{\mathbf{Gd}}$. $\chi_{\mathrm{M}}T$ is 7.48 cm^3 K mol^{-1} at 300 K and 1.68 cm^3 K mol^{-1} at 2 K. The red points represent a fit of the data according to equation 4 in the main text. Right: $M(H)$ data at 2 K.

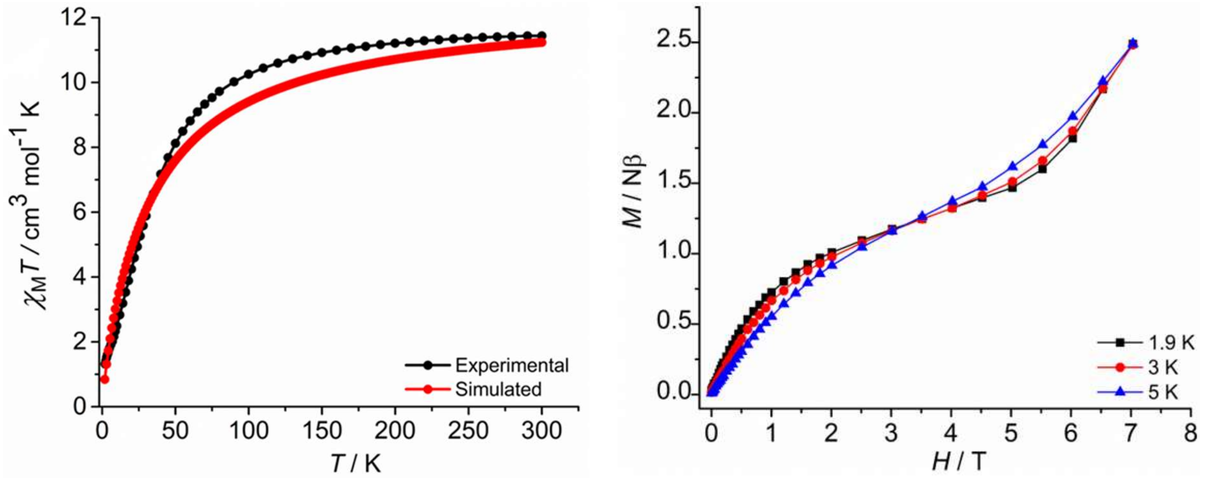

Figure S14. Left: $\chi_{\mathrm{M}}T(T)$ for **2**$_{\mathbf{Tb}}$. $\chi_{\mathrm{M}}T$ is 11.43 cm^3 K mol^{-1} at 300 K and 1.29 cm^3 K mol^{-1} at 2 K. The red points represent a fit of the data according to equation 5 in the main text. Right: $M(H)$ data at 1.9, 3.0 and 5.0 K.

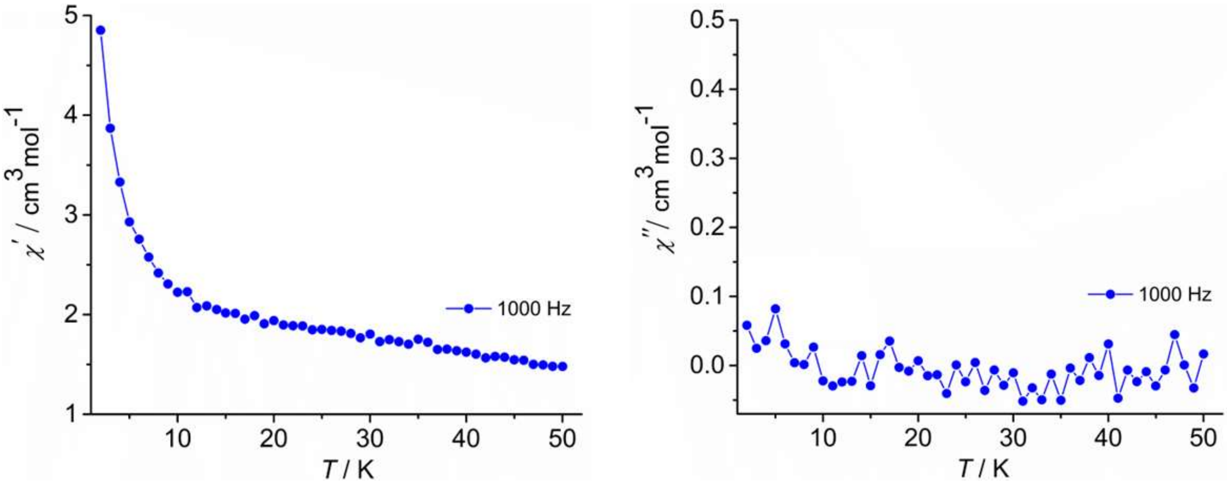

Figure S15. Real (left) and imaginary (right) components of the AC susceptibility as a function of temperature at 1000 Hz frequency in an AC field of 3 Oe and zero DC field for $\mathbf{2_{Tb}}$.

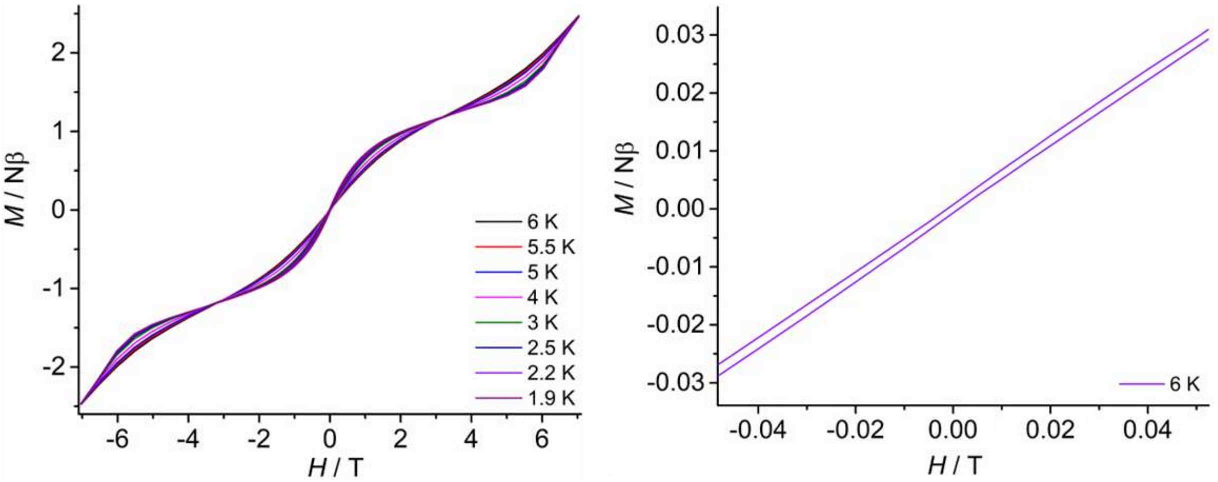

Figure S16. Magnetic hysteresis plot for $\mathbf{2_{Tb}}$. The data were continuously collected from 1.9 K to 6 K. under the following field sweep rates: 5 mT s^{-1} |0-0.02| T; 10 mT s^{-1} |0.02-0.1| T; 50 mT s^{-1} |0.1-0.40| T; 100 mT s^{-1} |0.4-2.0| T; 200 mT s^{-1} |2.0-3.0| T; 500 mT s^{-1} |3.0-5.0| T.

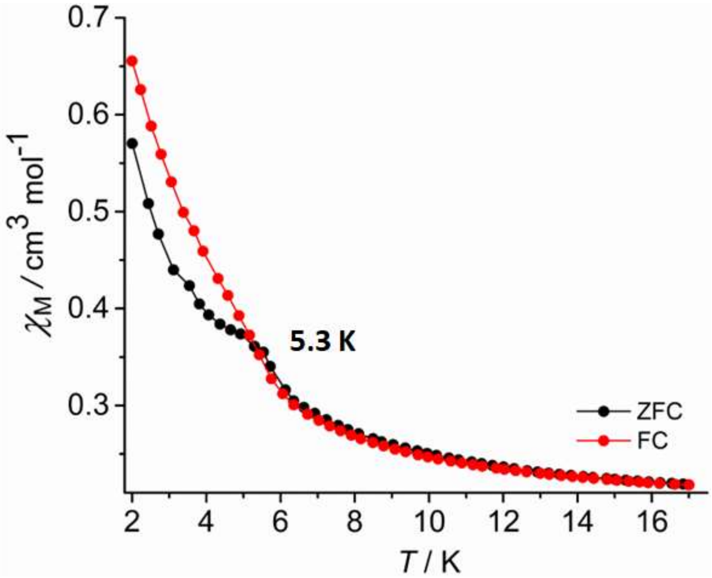

Figure S17. Zero-field-cooled (ZFC) and field-cooled (FC) magnetic susceptibility for $\mathbf{2_{Tb}}$ (scan rate = 1.6 K min^{-1}).

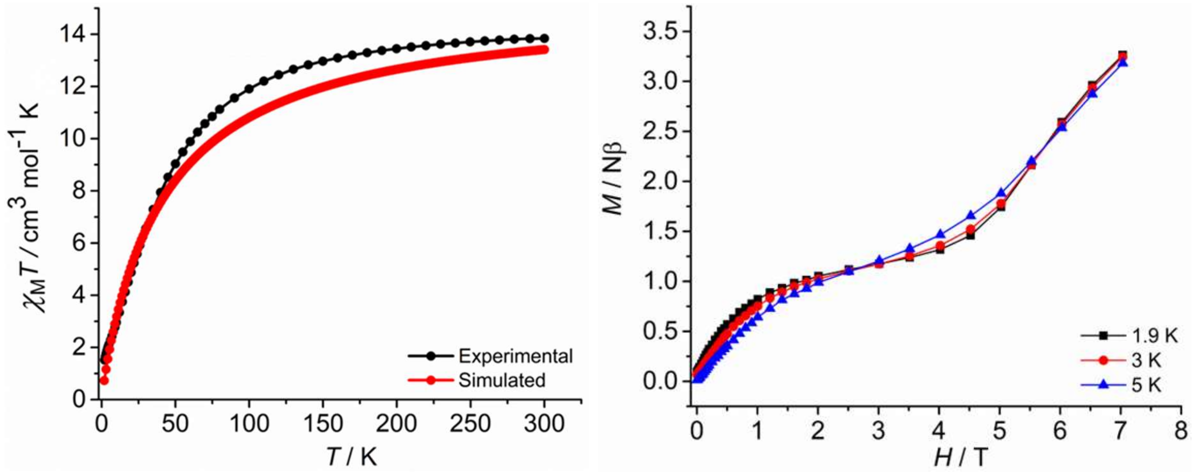

Figure S18. Left: $\chi_{\mathrm{M}}T(T)$ for $\mathbf{2_{Dy}}$. $\chi_{\mathrm{M}}T$ is 13.96 cm^3 K mol^{-1} at 300 K and 1.51 cm^3 K mol^{-1} at 2 K. The red points represent a fit of the data according to equation 5 in the main text. Right: $M(H)$ data at 1.9, 3.0 and 5.0 K.

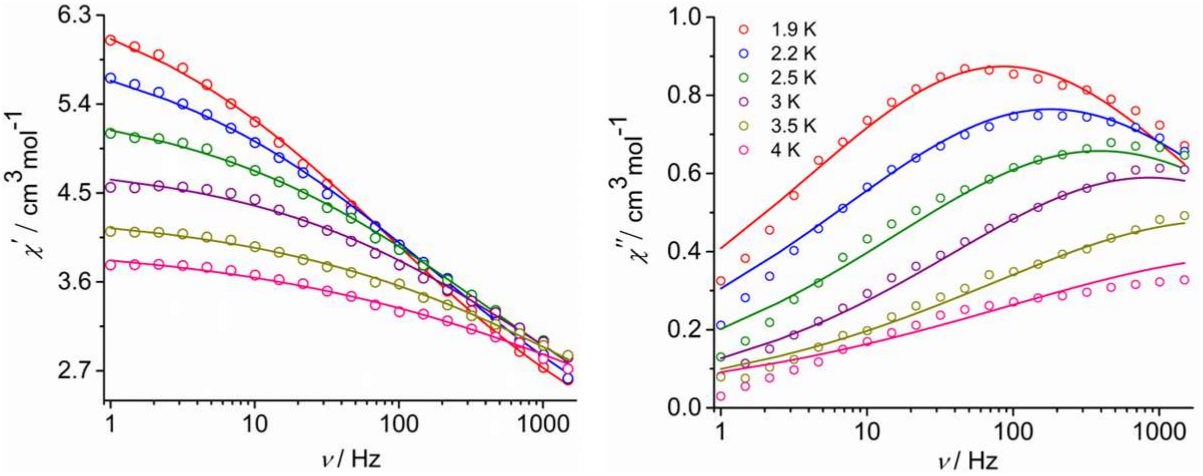

Figure S19. Real (left) and imaginary (right) components of the AC susceptibility as a function of frequency at T = 1.9-4 K in an AC field of 3 Oe and zero DC field for $\mathbf{2}_{\mathbf{Dy}}$.

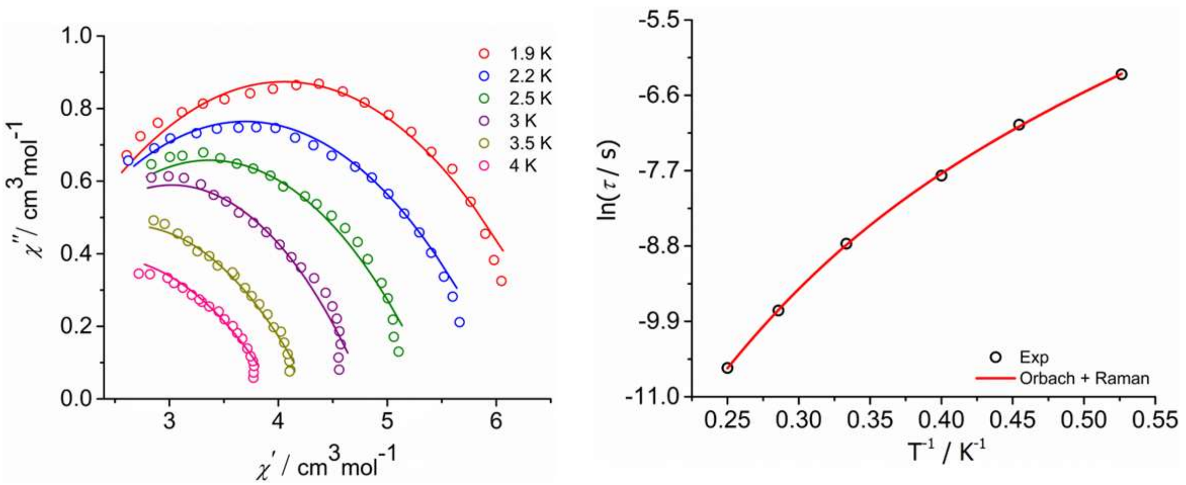

Figure S20. Cole-Cole plot (left) and temperature-dependence of the relaxation time (right) for $\mathbf{2}_{\mathbf{Dy}}$, where solid lines are fits to the data according to equations S2 and S3 and the parameters in Table S10.

Table S10. Relaxation fitting parameters for **2_{Dy}** for the Cole-Cole plot in Figure S20.

***T* / K**	**χ_S / cm^3 mol^{-1}**	**χ_T / cm^3 mol^{-1}**	**τ / s**	**α**
1.9	1.36614	6.73799	0.00185	0.59946
2.2	1.24153	6.15387	8.86654E-4	0.61583
2.5	1.27256	5.46046	4.21314E-4	0.61256
3.0	1.2179	4.82354	1.56034E-4	0.5978
3.5	0.93588	4.31778	5.881E-5	0.6488
4.0	0.80658	4.00802	2.54352E-5	0.69591

Fitting of the temperature-dependence of the relaxation times for **2_{Dy}** was achieved with the following parameters:

$$\tau^{-1} = \tau_0^{-1} exp^{(-\frac{U_{eff}}{k_B T})} + CT^n$$

C	19.23 ± 3.97
n	5.18 ± 0.3
U_{eff}	24 ± 5 cm^{-1}
τ_0	1.40 × 10^{-8} s

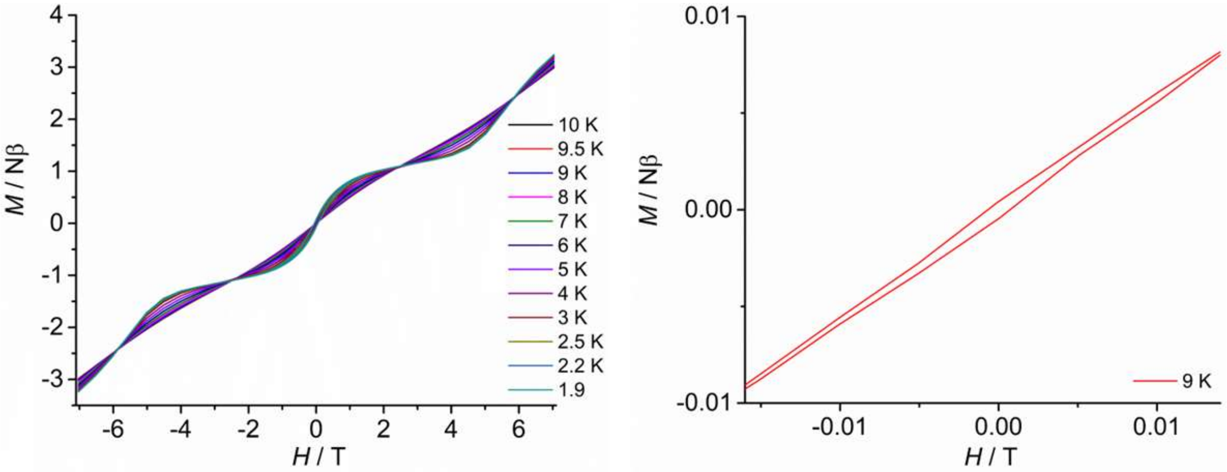

Figure S21. Magnetic hysteresis plot for **2_{Dy}**. The data were continuously collected from 1.9 K to 10 K. under the following field sweep rates: 5 mT s^{-1} |0-0.02| T; 10 mT s^{-1} |0.02-0.1| T; 50 mT s^{-1} |0.1-0.40| T; 100 mT s^{-1} |0.4-2.0| T; 200 mT s^{-1} |2.0-3.0| T; 500 mT s^{-1} |3.0-5.0| T.

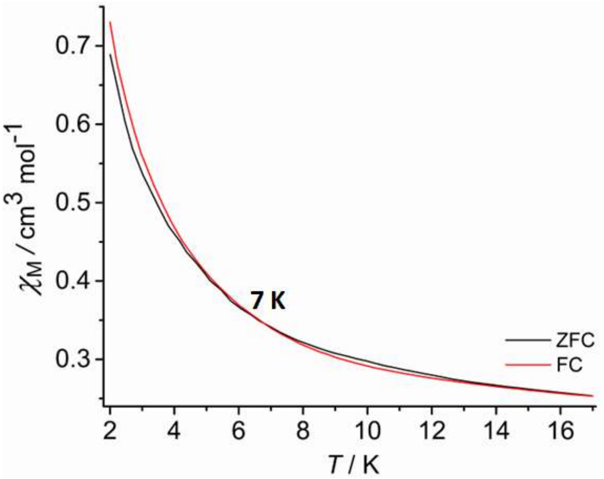

Figure S22 Zero-field-cooled (ZFC) and field-cooled (FC) magnetic susceptibility for $\mathbf{2_{Dy}}$ (scan rate = 1.6 K min^{-1}).

Ab initio calculations

All calculations were carried out on the coordinates obtained from the relevant crystal structure using the ORCA 5.0.2 software package. The positions of hydrogen atoms were optimized at the DFT level using a pure GGA PBE exchange correlation functional, keeping the position of the other atoms constant. The DKH Hamiltonian was used throughout to consider relativistic effects. The lanthanide centre was modelled with the SARC2-DKH-QZVP basis set, and all other atoms were treated with the DKH-def2-TZVP basis set in combination with the 'AutoAux' auxiliary basis set.[12] The active space CAS(8,7) was constructed from eight electrons in seven f-orbitals for terbium and CAS(9,7) was constructed from nine electrons in seven f-orbitals for dysprosium. In the configuration interaction procedure, seven septets, 76 quintets, and 52 triplets were computed for terbium whereas 21 sextets, 128 quartets, and 130 doublets were considered for dysprosium. To include the spin-orbit coupling, we also used the quasi-degenerate perturbation theory (QDPT) approach using SA-CASSCF wave functions.[13] The SINGLE_ANISO module as implemented in ORCA was used to compute the *g*-tensor and crystal field parameters of the low-lying excited states using previously calculated spin-orbit states.[14]

Table S11. SINGLE_ANISO computed crystal-field parameters B_k^q for **1**$_{\mathbf{Tb}}$ and **1**$_{\mathbf{Dy}}$.

k	***q***	**1**$_{\mathbf{Tb}}$	**1**$_{\mathbf{Dy}}$
	-2	-0.4296E+01	-0.5646E+00
	-1	0.2598E+00	-0.1252E+00
2	0	-0.6520E+01	-0.3640E+01
	1	0.4292E-01	-0.5560E-01
	2	0.1007E+02	0.6838E+01
	-4	-0.3039E-01	-0.3791E-02
	-3	-0.1528E-02	0.6835E-03
	-2	-0.2622E-02	-0.3865E-02
	-1	-0.4587E-02	0.8438E-03
4	0	0.8097E-02	-0.2420E-02
	1	-0.2178E-02	0.1598E-04
	2	-0.1438E-01	-0.6535E-02
	3	-0.1057E-01	-0.2317E-03
	4	0.8585E-02	-0.7707E-02
	-6	0.5862E-03	0.2516E-04
	-5	0.3458E-04	-0.2345E-04
	-4	-0.1086E-03	-0.2757E-04
	-3	-0.2049E-04	0.1526E-04
	-2	0.2946E-04	0.4296E-04
	-1	-0.1725E-04	0.1461E-04
6	0	-0.1458E-04	-0.4750E-05
	1	0.1909E-05	-0.8107E-04
	2	0.1276E-03	-0.3451E-04
	3	-0.5122E-05	-0.1009E-05
	4	0.2042E-04	-0.8107E-04
	5	0.1011E-03	0.2775E-04
	6	0.9576E-05	0.3455E-03

The Hamiltonian in equation S4 was used to calculate the crystal field parameters:

$$\hat{H}_{\mathrm{CF}} = \sum \sum_{k=-q}^{+q} B_k^q \hat{O}_k^q \quad \text{(S4)}$$

In equation S4, B_k^q is the crystal field parameter and $\hat{O}_k^q$ is the extended Stevens operator, where k is the rank of the irreducible tensor operator (ITO) ($k = 2, 4, 6$) and q is the component of the ITO ($q = -k, -k+1, \dots, 0, 1, \dots, k$).

Table S12. Ab initio calculated low-lying spin-orbit energy states for $\mathbf{1_{Dy}}$ and $\mathbf{1_{Tb}}$.

$\mathbf{1_{Tb}}$	$\mathbf{1_{Dy}}$
0.000	0.000
1.0618	0.000
107.4118	145.5162
115.2534	145.5162
224.1236	218.9870
251.8920	218.9870
306.9068	339.1481
416.8254	339.1481
428.2914	469.8643
687.3484	469.8643
687.9311	592.8744
1033.6793	592.8744
1033.6994	736.7218
2102.3748	736.7218
2125.8792	993.8245
2154.6213	993.8245

Table S13. Computed energies, tunnel splittings, g-tensors and wavefunction composition for the first six low-lying states in $\mathbf{1_{Tb}}$.

State	Energy/cm^{-1}	Tunnel splitting/cm^{-1}	g_z	Wave function composition
1	0.000	1.062	17.35	91.51%|±6>+7.83%|±4>+0.58%|±2
2	1.062			92.53%|±6>+7.15%|±4>+0.30%|±2
3	107.412	7.841	13.56	80.46%|±5>+17.04%|±3>+2.26%|±1
4	115.253			84.38%|±5>+14.15%|±3>+1.24%|±1
5	224.124	27.76	9.50	6.41%|±6>0.31%|±5>+50.95%|±4>+32.11%|±2 >+10.08%|0
6	251.892			6.87%|±6>0.39%|±5>+67.71%|±4>+22.93%|±2 >+1.90%|0

Table S14. Kramers doublet (KD) energies, g-tensors, angle between the anisotropic axis of the excited states and ground state, and wavefunction compositions for $\mathbf{1_{Dy}}$.

KD	E / cm^{-1}	g_x	g_y	g_z	Angle/°	Wave function composition
1	0.000	0.090	0.184	19.65	-	96.85%|±15/2>+2.81%|±11/2>
2	145.516	2.501	5.153	12.90	70.65	1.35%|±15/2>+35.0%|±13/2>+3.87%|±11/2>+17.32%|±9/2>+6.84%|±7/2>+14.09%|±5/2>+9.45%|±3/2>+12.04%|±1/2>
3	218.987	0.875	2.897	6.255	88.30	1.80%|±15/2>+42.82%|±13/2>+17.66%|±11/2>+1.48%|±9/2>+17.13%|±7/2>+1.16%|±5/2>+11.97%|±3/2>+5.95%|±1/2>
4	339.148	0.748	1.647	9.170	89.57	0.94%|±15/2>+16.07%|±13/2>+37.46%|±11/2>+11.25%|±9/2>+5.95%|±7/2>+18.52%|±5/2>+0.71%|±3/2>+9.05%|±1/2>
5	469.864	0.401	0.460	11.80	89.87	0.16%|±15/2>+4.61%|±13/2>+25.62%|±11/2>+29.56%|±9/2>+5.83%|±7/2>+5.67%|±5/2>+22.60%|±3/2>+5.91%|±1/2>
6	592.874	0.051	0.072	14.38	90.07	0.032%|±15/2>+0.09%|±13/2>+9.05%|±11/2>+25.68%|±9/2>+28.51%|±7/2>+8.03%|±5/2>+2.38%|±3/2>+25.51%|±1/2>
7	736.722	0.004	0.005	17.25	90.17	0.009%|±15/2>+0.30%|±13/2>+2.85%|±11/2>+11.65%|±9/2>+26.00%|±7/2>+33.60%|±5/2>+22.14%|±3/2>+3.42%|±1/2>
8	993.825	0.000	0.000	19.82	90.07	0.65%|±11/2>+3.0%|±9/2>+8.87%|±7/2>+18.82%|±5/2>+30.33%|±3/2>+38.23%|±1/2>

Table S15. SINGLE_ANISO computed crystal-field parameters B_k^q for [**2**$_{\mathbf{Tb}}$]$^+$ and [**2**$_{\mathbf{Dy}}$]$^+$.

k	***q***	**[2$_{\mathbf{Tb}}$]$^+$**	**[2$_{\mathbf{Dy}}$]$^+$**
	-2	0.2301E+01	0.2243E-03
	-1	0.1439E+00	-0.6748E-03
2	0	-0.4791E+01	-0.2455E+01
	1	0.1306E+01	0.7777E-00
	2	0.1033E+02	0.5631E+01
	-4	-0.1795E-01	-0.5381E-05
	-3	-0.1936E-01	-0.3848E-05
	-2	-0.1376E-02	0.1406E-05
	-1	-0.1462E-02	0.6488E-05
4	0	0.6227E-02	-0.1619E-02
	1	-0.1346E-01	-0.3092E-02
	2	-0.8208E-02	-0.5081E-02
	3	-0.5638E-01	-0.2280E-01
	4	-0.3839E-01	0.2022E-01
	-6	-0.4392E-03	-0.9544E-07
	-5	-0.3576E-03	-0.2997E-06
	-4	0.3516E-04	0.8652E-08
	-3	0.1463E-04	-0.9648E-07
	-2	-0.3433E-04	0.4353E-07
	-1	-0.2486E-04	-0.2690E-07
6	0	0.1767E-06	-0.1616E-04
	1	-0.2172E-03	-0.1222E-03
	2	-0.1771E-03	0.2015E-03
	3	0.5320E-04	-0.1017E-03
	4	0.7964E-04	0.3997E-04
	5	-0.5828E-03	-0.2401E-03
	6	-0.5703E-03	0.3456E-03

Table S16. Ab initio calculated low-lying spin-orbit energy states for hypothetical [$\mathbf{2}_{\mathbf{Tb}}$]$^+$ and [$\mathbf{2}_{\mathbf{Dy}}$]$^+$.

[$\mathbf{2}_{\mathbf{Tb}}$]$^+$	[$\mathbf{2}_{\mathbf{Dy}}$]$^+$
0.000	0.0000
11.9147	0.0000
24.6549	126.946
61.0324	126.946
96.0989	171.690
181.4782	171.690
196.3190	253.809
364.3006	253.809
366.9748	315.409
626.9388	315.409
627.3831	385.499
816.7561	385.499
816.8093	507.367
2042.0012	507.367
2052.4033	825.273
2123.4092	825.273

Table S17. Computed energies, tunnel splittings, g-tensors and wavefunction composition for first six low-lying states in [$\mathbf{2}_{\mathbf{Tb}}$]$^+$.

State	Energy/cm^{-1}	Tunnel splitting/cm^{-1}	g_z	Wave function composition
1	0.000	11.91	15.13	60.84%|±6>+22.32%|±4+11.55%|±2
2	11.915			80.53%|±6>+16.11%|±4

Table S18. Kramers doublet (KD) energies, g-tensors and wavefunction compositions for [$\mathbf{2_{Dy}}$]$^+$.

KD	*E* / cm^{-1}	g_x	g_y	g_z	Wave function composition
1	0.000	0.072	0.165	19.31	90.68%|±15/2>+8.21%|±11/2>0.38%|±9/2>+0.12%|±3/2>
2	126.946	2.073	6.153	13.53	0.90%|±15/2>+25.40%|±13/2>+1.72%|±11/2>+17.46%|±9/2>+10.10%|±7/2>+13.91%|±5/2>+14.88%|±3/2>+15.60%|±1/2>
3	171.691	2.677	4.460	7.73	1.15%|±15/2>+50.77%|±13/2>+4.16%|±11/2>+5.84%|±9/2>+9.74%|±7/2>+6.40%|±5/2>+10.49%|±3/2>+11.42%|±1/2>
4	253.809	3.793	5.904	8.265	4.50%|±15/2>+5.77%|±13/2>+40.65%|±11/2>+8.06%|±9/2>+17.11%|±7/2>+12.65%|±5/2>+4.56%|±3/2>+6.66%|±1/2>
5	315.409	1.675	2.018	11.46	1.35%|±15/2>+9.25%|±13/2>+19.09%|±11/2>+20.37%|±9/2>+9.28%|±7/2>+16.02%|±5/2>+17.58%|±3/2>+7.02%|±1/2>
6	385.499	0.034	0.059	14.60	0.91%|±15/2>+5.65%|±13/2>+16.73%|±11/2>+27.14%|±9/2>+18.80%|±7/2>+5.88%|±5/2>+6.60%|±3/2>+18.26%|±1/2>
7	507.367	0.032	0.033	17.28	0.42%|±15/2>+2.52%|±13/2>+8.03%|±11/2>+17.01%|±9/2>+25.99%|±7/2>+27.23%|±5/2>+16.23%|±3/2>+2.52%|±1/2>
8	825.273	0.000	0.000	19.81	0.38%|±13/2>+1.36%|±11/2>+3.70%|±9/2>+8.73%|±7/2>+17.79%|±5/2>+29.56%|±3/2>+38.38%|±1/2>

References

1. Cendrowski-Guillaume, S. M.; Le Gland, G.; Nierlich, M.; Ephritikhine, M. Lanthanide Borohydrides as Precursors to Organometallic Compounds. Mono(cyclooctatetraenyl) Neodymium Complexes. *Organometallics*, **2000**, 19, 5654–5660.
2. Venier, C. G.; Casserly, E. W. Di-tert-butylcyclopentadiene and tri-tert-butylcyclopentadiene. *J. Am. Chem. Soc.*, **2002**, 112, 2808–2809.
3. Goodwin, C. A. P.; Reta, D.; Ortu, F.; Liu, J.; Chilton, N. F.; Mills, D. P. Terbocenium: completing a heavy lanthanide metallocenium cation family with an alternative anion abstraction strategy. *Chem. Commun.*, **2018**, 54, 9182-9185.
4. Dolomanov, O. V.; Bourhis, L. J.; Gildea, R. J.; Howard, J. A. K.; Puschmann H. OLEX2: A Complete Structure Solution, Refinement and Analysis Program. *J. Appl. Cryst.*, **2009**, 42, 339–341.
5. Sheldrick, G. M. Crystal Structure Refinement with SHELXT. *Acta Cryst.*, **2015**, 71, 3–8.
6. Becke, A. D. Density-Functional Thermochemistry. III. The Role of Exact Exchange. *J. Chem. Phys.*, 1993, **98**, 5648-5652.
7. Dolg, M.; Stoll. H.; Preuss, H. Energy-Adjusted Ab initio Pseudopotentials for the Rare Earth Elements. *J. Chem. Phys.*, 1989, **90**, 1730-1734.
8. Schäfer, A.; Huber, C.; Ahlrichs, R. Fully Optimized Contracted Gaussian Basis Sets of Triple Zeta Valence Quality for Atoms Li to Kr. *J. Chem. Phys.,* 1994, **100**, 5829-5835.
9. Perdew, J. P.; Ernzerhof, M.; Burke, K. Rationale for Mixing Exact Exchange with Density Functional Approximations. *J. Chem. Phys.*, 1996, **105**, 9982−9985.
10. Neese, F.; Wennmohs, F.; Becker, U.; Riplinger, C. The ORCA Quantum Chemistry Program Package. *J. Chem. Phys.*, 2020, **152**, 224108.
11. Bain, G. A.; Berry, J. F. Diamagnetic Corrections and Pascal's Constants. *J. Chem. Educ*. **2008**, *85*, 532-536.
12. (a) Aravena, D.; Neese, F.; Pantazis, D. A. Improved Segmented All-Electron Relativistically Contracted Basis Sets for the Lanthanides. *J. Chem. Theory Comput.*, 2016, **12**, 1148-1156. (b) Chmela, J.; Harding, M. E. Optimized Auxiliary Basis Sets for Density Fitted Post-Hartree-Fock Calculations of Lanthanide Containing Molecules. *Mol. Phys.*, 2018, **116**, 1523-1538. (c) Weigend, F.; Ahlrichs, R. Balanced Basis Sets of Split Valence, Triple Zeta Valence and Quadruple Zeta Valence Quality for H to Rn: Design and Assessment of Accuracy. *Phys. Chem. Chem. Phys.*, 2005, **7**, 3297-3305.
13. Ganyushin, D.; Neese, F. First-Principles Calculations of Zero-Field Splitting Parameters. *J. Chem. Phys.*, 2006, **125**, 24103.
14. Chibotaru, L. F.; Ungur, L. Ab initio Calculation of Anisotropic Magnetic Properties of Complexes. I. Unique Definition of Pseudospin Hamiltonians and their Derivation. *J. Chem. Phys.*, 2012, **137**, 64112.
